# The meaning of ubiquitylation of the DSL ligand Delta for the development of *Drosophila*

**DOI:** 10.1186/s12915-023-01759-z

**Published:** 2023-11-16

**Authors:** Tobias Troost, Ekaterina Seib, Alina Airich, Nicole Vüllings, Aleksandar Necakov, Stefano De Renzis, Thomas Klein

**Affiliations:** 1 Institute of Genetics, Heinrich-Heine-Universitaet Duesseldorf, Universitaetsstr. 1, 40225 Duesseldorf, Germany; 2 Department of Biological Science, Brock University, 1030, Ontario, L2S3A1 Canada; 3 European Molecular Biology Laboratory, Developmental Biology Unit, Meyerhofstrasse 1, 69117 Heidelberg, Germany

**Keywords:** Cell communication, Notch, Delta, DSL-ligands, Ubiquitylation, E3-ligase, Mindbomb1, Neuralized, Endocytosis

## Abstract

**Background:**

Ubiquitylation (ubi) of the intracellular domain of the Notch ligand Delta (Dl) by the E3 ligases Neuralized (Neur) and Mindbomb1 (Mib1) on lysines (Ks) is thought to be essential for the its signalling activity. Nevertheless, we have previously shown that DlK2R-HA, a Dl variant where all Ks in its intracellular domain (ICD) are replaced by the structurally similar arginine (R), still possess weak activity if over-expressed. This suggests that ubi is not absolutely required for Dl signalling. However, it is not known whether the residual activity of DlK2R-HA is an effect of over-expression and, if not, whether DlK2R can provide sufficient activity for the whole development of *Drosophila*.

**Results:**

To clarify these issues, we generated and analysed *Dl*^*attP*^-*DlK2R-HA*, a knock-in allele into the Dl locus. Our analysis of this allele reveals that the sole presence of one copy of *Dl*^*attP*^-*DlK2R-HA* can provide sufficient activity for completion of development. It further indicates that while ubi is required for the full activity of Dl in Mib1-dependent processes, it is not essential for Neur-controlled neural development. We identify three modes of Dl signalling that are either dependent or independent of ubi. Importantly, all modes depend on the presence of the endocytic adapter Epsin. During activation of Dl, direct binding of Epsin appears not to be an essential requirement. In addition, our analysis further reveals that the Ks are required to tune down the cis-inhibitory interaction of Dl with Notch.

**Conclusions:**

Our results indicate that Dl can activate the Notch pathway without ubi of its ICD. It signals via three modes. Ubi is specifically required for the Mib1-dependent processes and the adjustment of cis-inhibition. In contrast to Mib1, Neur can efficiently activate Dl without ubi. Neur probably acts as an endocytic co-adapter in addition to its role as E3 ligase. Endocytosis, regulated in a ubi-dependent or ubi-independent manner is required for signalling and also suppression of cis-inhibition. The findings clarify the role of ubi of the ligands during Notch signalling.

**Supplementary Information:**

The online version contains supplementary material available at 10.1186/s12915-023-01759-z.

## Background

Many developmental, homeostatic and pathological processes in metazoans depend on signalling through the Notch pathway [1]. Canonical Notch signalling is initiated by transmembrane proteins of the DSL family, Delta (Dl) and Serrate (Ser) in *Drosophila*, and is thought to require endocytosis of the ligands interacting in trans with the Notch receptor [2, 3]. This endocytic event depends on the endocytic adapter Epsin and creates a pulling force that induces a conformational change in Notch [4–8]. The change in conformation exposes a cleavage site cleaved by ADAM10 (S2 cleavage) to create a truncated, membrane inserted intermediate that is immediately cleaved by the γ -secretase (S3/4 cleavage). This sequence of cleavages results in the release of the Notch intracellular domain (NICD) into the cytosol. After being transported to the nucleus, NICD contributes to the formation of an activator complex formed around the CSL transcription factor Suppressor of Hairless (Su(H)) to initiate target gene expression. The cleaved off NECD remains attached to the ligand and is trans-endocytosed into the signal-emitting cell [9].

Previous work on the *Drosophila* ligand Delta (Dl) has revealed that it is internalised by two different endocytosis pathways, termed bulk and Epsin-dependent endocytosis [3, 10]. Bulk endocytosis is by far the prevailing pathway, but irrelevant for signalling. The Epsin-dependent pathway is instrumental for signalling, but comprises only a small fraction of endocytosed Dl, which was revealed only by over-expression experiments [5, 10]. Recent work indicates that only the Epsin pathway appears to generate the necessary pulling force level in the endocytic pit to induce the conformational change in Notch [3, 5, 6, 8].

Endocytosis of transmembrane proteins is often initiated by E3-ligase-mediated ubiquitylation (ubi) of lysines (Ks) of their intracellular domains (ICDs) [3, 11]. Two E3-ligases, termed Mindbomb1 (Mib1) and Neuralized (Neur), have been identified as relevant for signalling and ubi of Dl, as well as the other *Drosophila* DSL ligand Serrate (Ser) [3, 12–16]. Both ligases are involved in complementary Notch-dependent processes and bind to different epitopes of the ICD of Dl [17, 18]. Neur is required almost exclusively for neural developmental processes, such as the selection of neural precursor cells. Its loss of function results in a hyperplasia of the embryonic nervous system at the expense of epidermal development [19]. This neurogenic phenotype is characteristic for loss of function of many genes involved in Notch signalling. It causes the death of mutant flies at the end of embryogenesis with only a small patch of cuticle.

In the imaginal discs of *Drosophila*, Mib1 is ubiquitously expressed, whereas Neur is restricted to the single, late arising sensory organ precursor cells (SOPs) [14, 20]. Hence, most Notch signalling is mediated by Mib1. The loss of function of *mib1* in the wing disc causes a severe reduction of Notch activity, which results in the loss of most parts of the wing [14]. However, Neur-dependent selection of SOPs is not affected [21]. Moreover, Mib1-dependent ubi of Dl is a prerequisite only for the Epsin-dependent pathway, but not for bulk endocytosis [3, 10]. Importantly, no obvious defects in endocytosis of Dl have been observed in *epsin* or *mib1* mutant cells [10, 14, 21]. Weak defects upon loss of *mib1* function were observed only under strong over-expression [10, 21].

The ubiquitylated Dl is recognised by the endocytic adapter Epsin, encoded by *liquid facets* (*lqf*) in *Drosophila*. Epsin binds to ubiquitylated Dl via its two ubiquitin interacting motifs (UIMs) to concentrate Dl in the nascent endocytic pit and is also involved in the induction of membrane curvature and also in the organisation of the actin cytoskeleton the forms around the endocytic pit. It is likely that the last two functions are essential for pulling force generation during Notch activation (reviewed in [3]).

Besides activating Notch in trans, the ligands can also engage in inhibitory interactions with the receptor in cis, a mechanism termed cis-inhibition (CI). CI has been shown to be employed in several developmental processes to regulate the activity of the pathway [22]. The critical parameter for CI is the ratio between ligand and receptor levels, shown by the finding that CI by the ligands can be suppressed by elevation of the levels of Notch [23–25]. The mechanism of CI is not understood in detail, but the DSL region of the extracellular domain (ECD) of the ligands is essential, suggesting a similar interaction of the ligand with Notch in cis and trans [26].

We have previously tested the requirement of ubi for the function of Dl by characterising a ubi-deficient variant where the Ks of its ICD are replaced by the structurally similar arginine (R) [27]. Surprisingly, this DlK2R variant, was able to induce weak Notch pathway activity, even in the absence of *mib1-* and *neur-*function. Altogether, this analysis suggested that, although ubi is required for activation of Dl by Mib1, Dl functions partly in an ubi-independent manner. A further interesting finding was that ubi of the ICD of Dl is important to suppress CI, as DlK2R possesses significantly higher cis-inhibitory abilities than Dl. Unexpectedly, we found that Neur acts in a different manner than Mib1: it can activate DlK2R-HA as good as Dl-HA, suggesting that ubi is of minor, or even no importance for the activation of Dl by Neur.

A caveat of this analysis is that it was conducted with Gal4-induced over-expression of DlK2R-HA. It is therefore possible that the residual, ubi-independent activity is only observed under this overexpression condition and irrelevant for Dl-signalling during development of *Drosophila*. Moreover, since the previous study was focussed on SOP and wing development, it is not known whether DlK2R-HA can provide sufficient activity in all Dl-dependent processes. To clarify these issues, we generated a DlK2R knock-in allele, where DlK2R-HA is expressed under the control of the endogenous *Dl* promoter. We found that the presence of already one copy of this DlK2R-HA allele in the genome can provide sufficient activity for the complete development of *Drosophila*. However, the emerging flies displayed weak Notch loss of function phenotypes in Mib1-dependent processes. The analysis shows that ubi appears to be important mostly for Mib1-regulated, non-neural processes that are mediated by Dl. Neur can efficiently activate Dl also in the absence of Ks and the neural Dl-regulated processes are therefore hardly affected. We found that Dl can signal in three ways, the Mib1- and ubi-dependent way, the Neur-mediated, ubi-independent way and the ubi-, Mib1- and Neur-independent way. Importantly, also the two ubi-independent ways depend on Epsin, suggesting that a physical interaction between Epsin and Dl is not absolutely necessary, or occurs in a so far unappreciated manner.

## Results

**Fig. 1:**
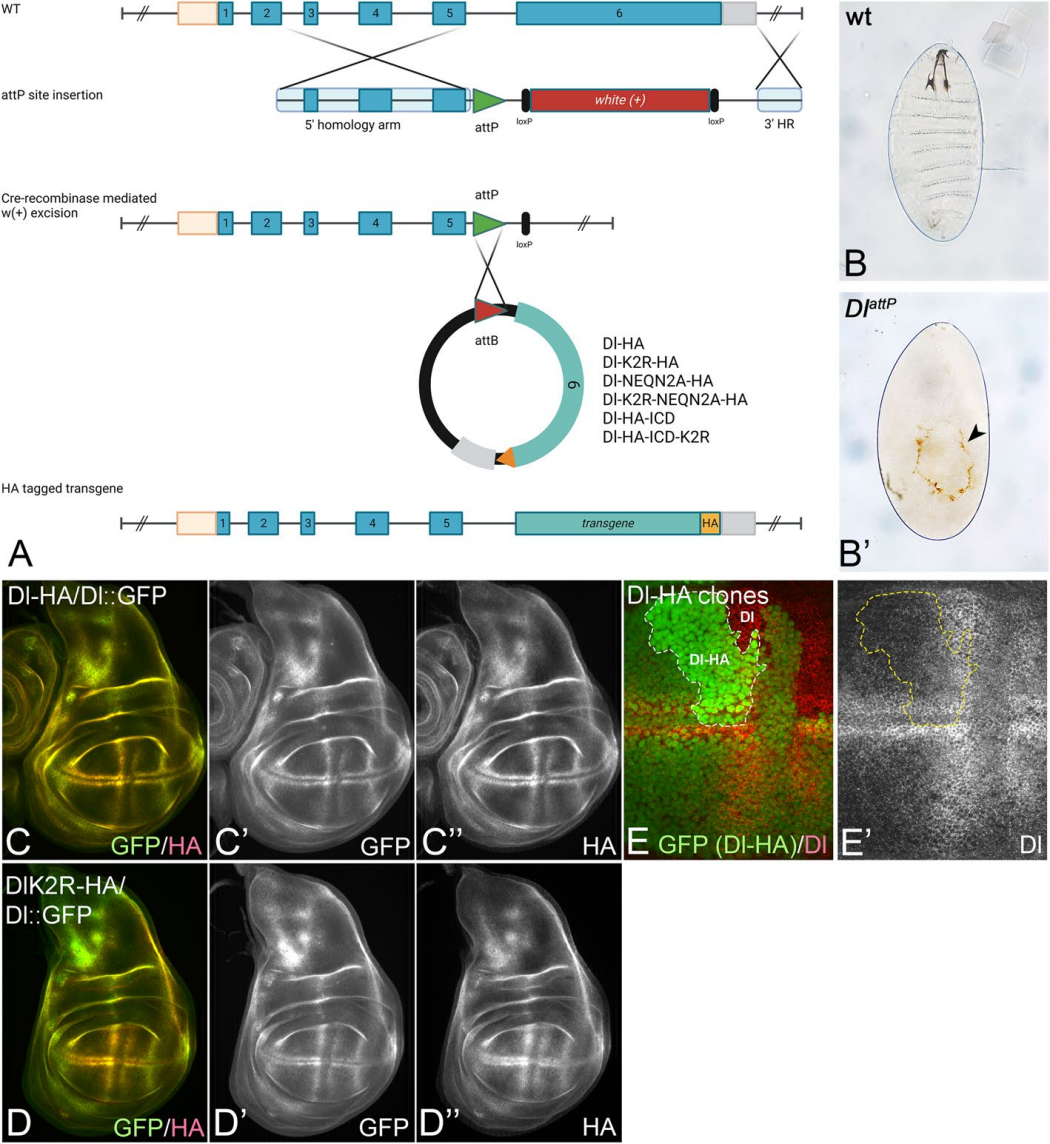
Generation of the *Dl*^*attP*^ alleles. **A** Generation and use of *Dl*^*attP*^. Exon 6 of Dl was replaced by an attP landing site. This replacement creates a *Dl* null mutant allele *Dl*^*attP*^. The attP site allows the insertion of modified exon6-variants. **B**, **B’** Cuticle preparations of wt (**B**) and *Dl*^*attP*^ (**B’**) embryos. The *Dl*^*attP*^ mutant flies possess only a small patch of dorsal cuticle (arrowhead in **B’**). This neurogenic phenotype is characteristic for mutants of genes encoding Notch pathway components. **C–D’’** Expression of Dl^attP^-Dl-HA (**C–C’’**) and Dl^attP^-DlK2R-HA (**D–D’’**) in comparison to Dl::GFP. **E, E’** The wing region of a wing disc bearing Dl-HA homozygous clones (bright green, Dl-HA). The clone is outlined in white and yellow. The clone is next to a clone homozygous for endogenous Dl (Dl, no green). **E’** The expression levels of both Dl-variants is indistinguishable, indicating that the knock-in of a HA-modified exon 6 does not affect the levels of expression

To answer the questions whether the residual activity of DlK2R is observed only under conditions of over-expression and, if not, whether it could provide sufficient activity for the complete development of *Drosophila*, we made use of a fly line where exon 6 of genomic *Dl* is replaced by an attP landing site (*Dl*^*attP*^) [28]. Exon 6 encodes most of Dl, including its ICD (Fig. 1A). *Dl*^*attP*^ is a true null allele, causing the death of homozygous flies during embryogenesis. They display the neurogenic cuticle phenotype characteristic for loss of function of genes encoding members of the Notch signalling pathway [19, 28] (Fig. 1B, B’). *Dl*^*attP*^ allows to generate knock-in alleles encoding variants, such as DlK2R, expressed under the endogenous *Dl*-promoter by insertion of a modified exon 6 into the *Dl*^*attP*^ landing site (Fig. 1A). We initially generated two variants, *Dl*^*attP*^*-Dl-HA* (control) and *Dl*^*attP*^*-DlK2R-HA*. The HA-tag allowed us to monitor the expression pattern of these variants by anti-HA antibody staining. To judge the correctness of their expression, we used the expression of Dl::GFP, a genome edited functional version of Dl fused to GFP, as a reference [29]. The comparison of the expression with Dl::GFP in the wing, leg and haltere imaginal discs, the larval brain, the embryo, as well as in the adult gut revealed that both Dl^attP^-variants are expressed in the correct pattern (Fig. 1C–D’’, Additional file 1: Fig. S1). To test whether the control Dl^attP^-Dl-HA allele is expressed at the correct levels, we compared its expression with endogenous Dl. To do so, we induced Dl^attp^-*Dl-HA* homozygous clones adjacent to clones homozygous  for endogenous (wildtype) *Dl* and performed anti-Dl antibody staining. We did not observe any differences in the levels of Dl in the two types of clones, indicating that the knock-in Dl-HA allele is correctly expressed. The result also indicates that the addition of the HA tag has no obvious effect on expression or subcellular localisation (Fig. 1E, E’).

### One copy of endogenously expressed DlK2R-HA produces sufficient activity to allow complete development of *Drosophila*

**Fig. 2:**
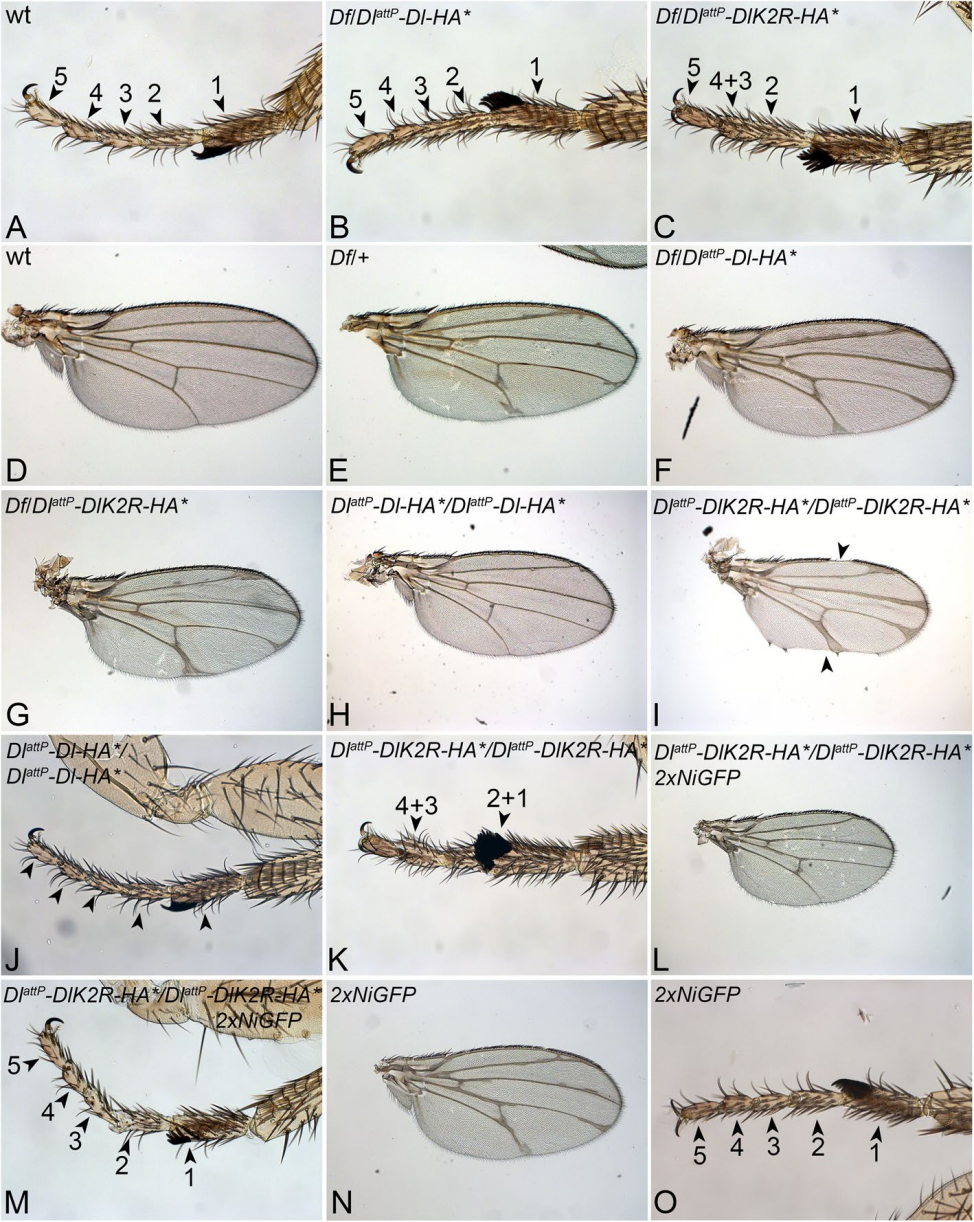
Analysis of the phenotype of adult *Dl*^*attP*^*-Dl-HA* and *Dl*^*attP*^*-DlK2R-HA* flies. **A**–**G** The phenotype of flies with one copy of *Dl*^*attP*^*-Dl-HA* (**B**, **F**) and *Dl*^*attP*^*-DlK2R-HA* (**C**, **G**) over the deficiency *Dl*^*BSC850*^ (*Df*). Compare with the wildtype control, shown in **A**, **D**. In contrast to the wildtype and *Dl*^*attP*^*-Dl-HA*/*Df* flies, the tarsal segments 4 and 3 are fused in *Dl*^*attP*^*-DlK2R-HA*/*Df* flies*.***E**, **F***Dl*^*attP*^*-Dl-HA*/*Df* flies show the typical haplo-insufficient wing vein broadening of *Dl*. The vein broadening is only slightly enhanced in *Dl*^*attP*^*-DlK2R-HA/Df* flies. **H**, **J** The phenotype of flies homozygous for *Dl*^*attP*^*-Dl-HA* resembles that of wildtype flies (compare with **A**, **D**). **I**, **K** In contrast, homozygous *Dl*^*attP*^*-DlK2R-HA* displays enhanced broadening of the wing veins and nicks in the wing margin (**I**, arrowheads). Moreover, tarsal segments 1 and 2 are fused in addition to 3 and 4 (**K**, arrowheads). **L**–**N** The phenotype of *Dl*^*attP*^*-DlK2R-HA* homozygous flies is suppressed if two additional copies of Notch are present

We first tested the effect of only one copy of the two Dl^attP^-variants on development by analysing them over the chromosomal deficiencies *Dl*^*BSC850*^, or *Dl*^*rev10*^ (*Df*), which both include the *Dl* locus. As expected, *Dl*^*attP*^*-Dl-HA*/*Df* control flies developed to adulthood and displayed the dominant haplo-insufficient phenotype characteristic for *Dl*-heterozygous flies (Fig. 2A, B, D–F). Thus, similar to endogenous *Dl*, one copy of *Dl*^*attP*^*-Dl-HA* is sufficient to allow the complete development, giving rise to flies with the expected phenotype.

To our surprise, *Dl*^*attP*^*-DlK2R-HA/Df* flies also developed to the adult stage and hatched. The adult flies displayed a phenotype that was only slightly stronger than the haplo-insufficient *Dl* phenotype. They had fusions of tarsal segments 3 and 4 and slightly enhanced broadening of the wing veins in comparison to *Dl*^*attP*^*-*Dl-HA/Df and Df/ + flies (Fig. 2A, C, D, G). Moreover, the *Dl*^*attP*^*-DlK2R-HA/Df* females produced only very few eggs, revealing a strong decrease in fertility.

Altogether, these results indicate that the sole presence of a single copy of *Dl*^*attP*^*-DlK2R-HA* in the genome allows the complete development of *Drosophila,* giving rise to vital, but nearly sterile flies, which display a surprisingly weak Notch loss of function phenotype. Complete loss of function of Dl results in embryonal lethality. Thus, Dl^attP^-DlK2R-HA can provide a significant amount of Dl-activity.

### The Ks in the ICD of Dl are required to suppress CI

As expected for a wildtype allele, homozygous *Dl*^*attP*^*-Dl-HA* flies resembled wildtype flies in all aspects monitored, indicating that an increase in copy number leads to an increase in activity of the Notch pathway and the suppression of the haplo-insufficient phenotype of *Dl*. (Fig. 2D, H, J). In contrast, homozygous *Dl*^*attP*^*-DlK2R-HA* flies displayed a more severe phenotype than *Dl*^*attP*^*-DlK2R-HA/Df* flies. The broadening of wing veins was significantly more enhanced and nicks occurred in the wing margin (Fig. 2K arrowheads, compare with G). Moreover, the tarsal segments 1 and 2 were fused in addition to 3 and 4 (Fig. 2K, compare with C). Hence, the increase in copy number of *Dl*^*attP*^*-DlK2R-HA* (homozygosity) caused a more severe, instead of the expected wildtype phenotype, indicating a reduction in Notch pathway activity.

Concentration-dependent suppression of Notch activity by the ligands is the hallmark of CI, as it depends on the ratio between the levels of Notch and its ligand [24, 30]. We have previously shown that Gal4 over-expressed DlK2R had increased cis-inhibitory abilities, which could be suppressed by an increase of the levels of Notch [27]. To test whether an increase in CI causes the enhancement of the phenotype of homozygous *Dl*^*attP*^*-DlK2R* flies, we increased the copy number of *Notch* by introducing a BAC that contains the genomic region of Notch. The BAC encodes a fully active NotchiGFP fusion protein (NiGFP) [31]. We found that the presence of two copies of NiGFP in the genome in addition to the endogenous copies, suppressed the phenotype of *DlK2R-HA* homozygous flies. The original number of five tarsal segments was restored and the broadening of the veins suppressed (Fig. 2L, M, O). In addition, no nicks in the margin were observed (Fig. 2L, I). Note that the combination mutually repressed the wing phenotypes of each individual genotype. Both individual genotypes displayed broadening of the wing veins (compare Fig. 2I, L, N). This finding confirms the finding of Berndt et al. [27] that Dl^attP^-DlK2R-HA has increased cis-inhibitory abilities.

### The Ks are required for effective endocytosis of Dl

**Fig. 3:**
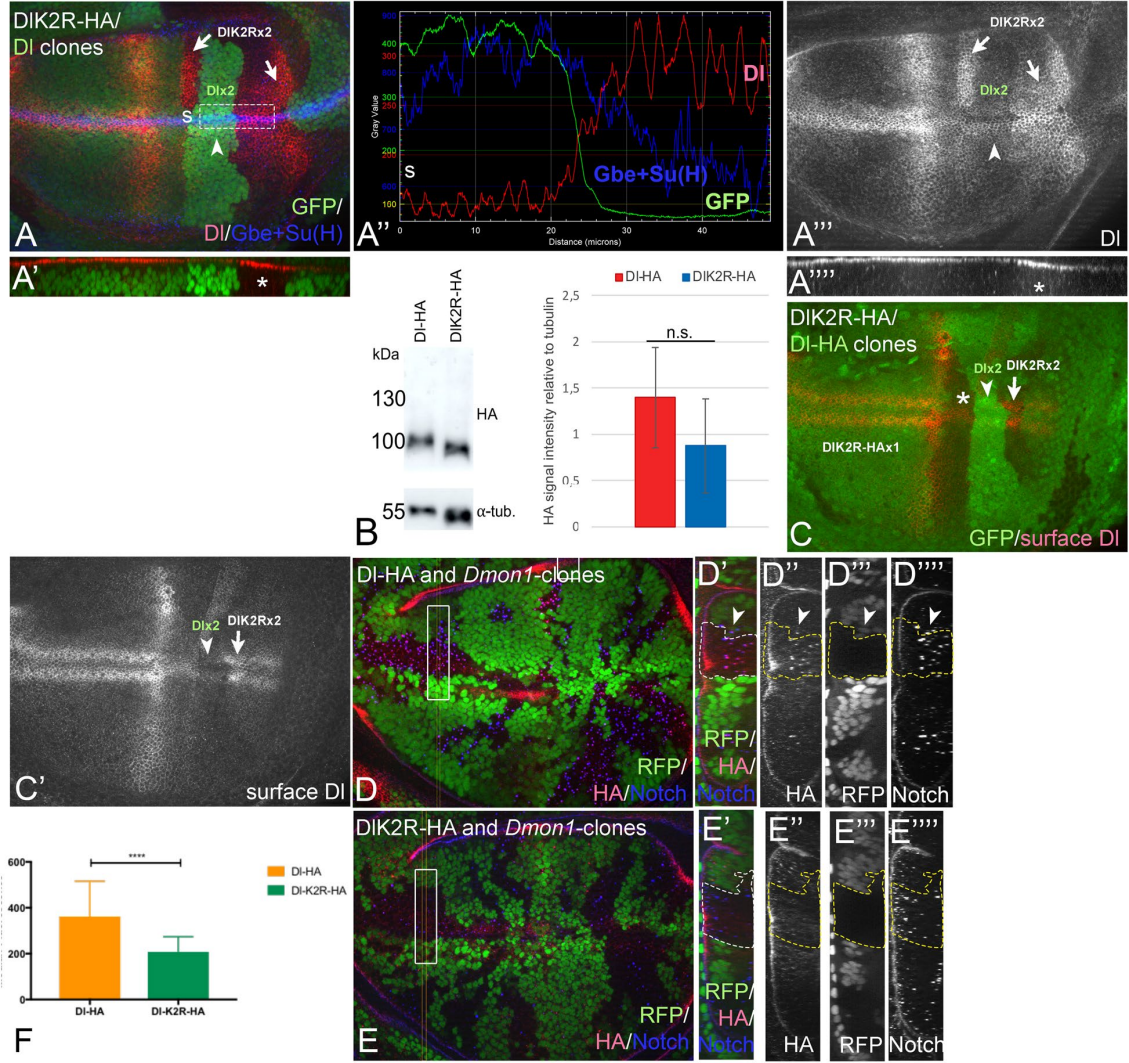
The Ks in the ICD of Dl are required for efficient endocytosis. **A–A’’** Clonal analysis of *Dl*^*attP*^*-DlK2R-HA*. The homozygous *Dl*^*attP*^*-DlK2R-HA* clones are labelled by loss of GFP and highlighted by the arrows. The comparison with the clone homozygous for endogenous Dl (arrowhead), reveals the higher abundance of DlK2R-HA in the apical plasma membrane of homozygous cells (arrows). See also the z-sections in **A’, A’’’’**, asterisk. **A’’** Pixel density measurement of the apical region highlighted in **A** with the rectangle (s: start-point of measurement). It shows the higher abundance of DlK2R-HA in the apical membrane. **B–B’’** Western-blot analysis of the Dl variants revealed that they are similarly expressed (*n* = 3). **C**, **C’** Detection of surface Dl by applying the primary antibody in the absence of detergence. In this clonal analysis, homozygous *Dl*^*attP*^*-Dl-HA* clones (dark green, arrowhead) adjacent to homozygous *Dl*^*attP*^*-DlK2R-HA* clones (arrow) were induced and the Dl-variants detected with anti-Dl antibody the binds to the ECD*.* The staining reveals that homozygous *Dl*^*attP*^*-Dl-HA* cells have less Dl on its surface than homozygous or heterozygous *Dl*^*attP*^*-DlK2R-HA* cells. **D–E’’’’** Wing discs where *Dmon1* and *Dl*^*attP*^*-Dl-HA-* (**D–D’’’’**) or *Dmon1* and *Dl*^*attP*^*-DlK2R-HA*- (**E–E’’’’**) clones were induced. **D, E** Overview of the disc bearing the clones. The homozygous *Dmon1Dl*^*attP*^*-variants* clones are labelled by the loss of GFP and highlighted by the arrow. **D’–D’’’’**, **E’–E’’’’** z-section of the regions highlighted in **D, E** by the rectangle. A double mutant clone is outlined in white or yellow. **F** Quantification of the association of the enlarged Notch-positive endosomes of *Dmon1* cells with the HA-signal (Dl-variants, *n* = 3 for each genotype, see M&M for details). It confirms less association of DlK2R-HA with the enlarged Notch positive endosomes compared to Dl-HA

Upon comparison of the expression of both Dl^attP^-variants, we had the impression that DlK2R-HA is more abundant in the plasma membrane than Dl-HA and Dl::GFP. To validate this impression, we used clonal analysis to directly compare the expression of DlK2R with that of endogenous untagged Dl in adjacent homozygous clones, as we have done for an Dl-HA (see Fig. 1D, D’). We found higher levels of Dl in Dl^attP^-DlK2R-HA compared to wt clones (endogenous Dl) in the apical plasma membrane (Fig. 3A-A’’’’). The higher abundance of DlK2R in the apical membrane was also observed when we directly compared the expression of Dl^attP^-DlK2R-HA with Dl^attP^-Dl-HA using clonal analysis (Additional file 1: Fig. S2A-B’’). Western Blot analysis revealed that both Dl^attP^-variants are expressed at similar levels in the cell (Fig. 3B). Therefore, the difference between DlK2R-HA and Dl-HA must be founded in different subcellular localisation and DlK2R is more abundant in the plasma membrane. To further confirm this notion, we performed antibody staining on discs where the cells were not permeabilised with detergents during the incubation with a primary antibody directed to the ECD of Dl. In this set up, only Dl inserted in the plasma membrane (surface Dl) is detected. The staining confirmed the higher abundance of DlK2R in the plasma membrane (Fig. 3C, C’, arrow and arrowhead). These results suggest that the loss of the Ks in the ICD results in an accumulation of Dl in the apical membrane.

In our clonal analysis shown in Fig. 3A-A’’’, the differences in the abundance of Dl were less significant if we compared heterozygous DlK2R/ + with homozygous DlK2R clone cells. We have previously shown that DlK2R is less efficiently endocytosed and degraded under over-expression conditions [27]. Thus, it is likely that the observed accumulation of Dl^attP^-DlK2R-HA in the plasma membrane is caused by its inefficient endocytosis. This accumulation of Dl^attP^-DlK2R-HA could explain the observed smaller difference between heterozygous and homozygous Dl^attP^-DlK2R-HA cells. To further confirm a defect in endocytosis, we compared the abundance of the variants in endosomes of *Dmon1* mutant cells. The loss of *Dmon1* function results in a failure of fusion of the endosomes with the lysosome [32, 33]. As a result, the lifetime of the endosomes is extended, they enlarge dramatically and accumulate cargo, such as Notch and Dl [33]. We found that fewer of the enlarged *Dmon1*-mutant endosomes contained Dl^attP^-DlK2R-HA compared to Dl^attP^-Dl-HA and that the signal associated with the endosomes is weaker, suggesting that DlK2R-HA does enter the endosomal pathway with less efficiency (Fig. 3D–F’’’).

Formally, the higher abundance of DlK2R in the apical membrane could also be caused by enhanced recycling after endocytosis. The main recycling routes are controlled by the small GTPases Rab11 and Rab4 (slow and fast recycling, respectively) [34]. We found that the concomitant efficient co-depletion of Rab11 and Rab4 did not affect the level of DlK2R in the plasma membrane of wing disc cells (Additional file 1: Fig. S2C-D’). Thus, it appears that the higher abundance of DlK2R in the plasma membrane is not caused by enhanced recycling. Our results are in line with previous work by Banks et al. [35]. Altogether, these findings suggest that the accumulation of DlK2R in the plasma membrane is a result of reduced endocytosis in comparison to Dl. Thus, the Ks of the ICD appear to contribute to the efficiency of endocytosis of Dl. However, it is worth to highlight that Dl^attP^-DlK2R-HA can still be detected in endosomes, confirming previous work that Dl is also endocytosed in a K- and ubi-independent manner [14, 21, 27].

### Signalling activity of the DlattP-variants

**Fig. 4:**
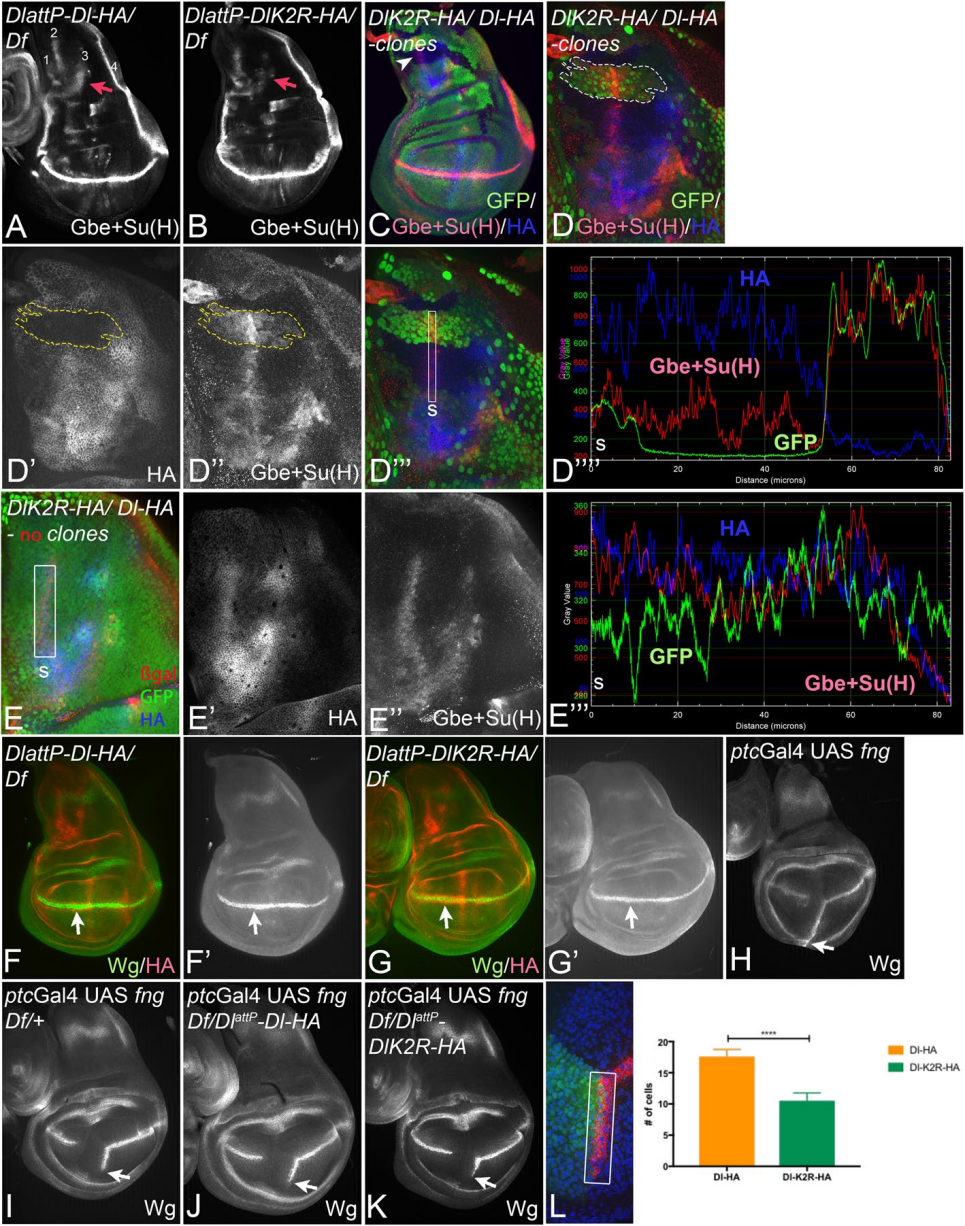
The Ks in its ICD are required for the full activity of Dl. **A**, **B** Gbe + Su(H) expression in *Dl*^*attP*^*-Dl-HA* and *Dl*^*attP*^*-DlK2R-HA*/*Df* discs. Four stripes are recognisable in the wt disc in **A**. The arrow points to stripe3 which is decreased in *Dl*^*attP*^*-DlK2R-HA/Df* discs. **C–D’’** A wing disc bearing homozygous *Dl*^*attP*^*-Dl-HA* (bright green outlined in white) *andDl*^*attP*^*-DlK2R-HA* (no green) twin clones*.***C** Overview. **D–D’’’’** Magnification of the notal area highlighted in **C** with the arrowhead. The homozygous Dl-HA clone is outlined in white or yellow. Although Dl^attP^-Dl-HA accumulates to lower levels than Dl^attP^-K2R-HA in the plasma membrane (**D’**), Gbe + Su(H) is stronger expressed in Dl^attP^-Dl-HA homozygous territory (**D’’**), indicating that Dl^attP^-Dl-HA can activate the Notch pathway more strongly and that also stripe2 is affected by the loss of the Ks in the ICD of Dl. **D’’’**, **D’’’’** pixel density measurement reveals the Gbe + Su(H) is increased in the clone homozygous for Dl-HA, whereas the HA-signal drops. The converse is true in the adjacent DlK2R-HA homozygous clone. s: start of the measurement. See also Additional file 1: Fig. S3 for more examples. **E–E’’’** Measurement in a control wing disc with the same genotype as in **C–D'’’’** without clones for comparison. **F**, **G’** Expression of Wg in *Dl*^*attP*^*-Dl-HA*/*DfandDl*^*attP*^*-DlK2R-HA*/*Df* discs. The arrow points to the expression of Wg along the D/V-boundary. **H–L** induction of ectopic Wg expression by expression of Fng with *ptc*Gal4. The length of the ectopic stripe of Wg expression is strongly reduced in *Dl*^*attP*^*-DlK2R-HA*/*Df* compared to *Dl*^*attP*^*-Dl-HA*/*Df* discs (arrow). **L** Quantification of the length of the ectopic stripe of Wg expression induced by Fng in the Dl^attP^ variants. The length is measured by the number of cells in the ectopic Wg stripe (rectangle, *n* = 12 for Dl-HA and *n* = 18 for DlK2R)

We next compared the signalling abilities of the two Dl^attP^-variants. We first monitored the global expression of the Notch activity reporter Gbe + Su(H) in the notum of wing imaginal discs*.* In this region, Gbe + Su(H) is expressed in four stripes [36] (Fig. 4A).

We observed a significant reduction in expression of stripe3 in *Dl*^*attP*^*-DlK2R-HA/Df*, compared to *Dl*^*attP*^*-Dl-HA/Df* notae (Fig. 4A, B, arrow). This reduction in Gbe + Su(H) expression indicates a significant reduction in the ability of *Dl*^*attP*^*-DlK2R-HA* to activate the Notch pathway.

Next, we compared the expression of Gbe + Su(H) in the described twin clone experiment where the twin clones are homozygous for one of the Dl^attP^-variants to test whether stripe2 expression of the Notch activity reporter Gbe + Su(H) is also reduced in *Dl*^*attP*^*-DlK2R-HA* homozygous cells. The expression in stripe2 was reduced in homozygous *Dl*^*attP*^*-DlK2R-HA* clones compared to the adjacent homozygous *Dl*^*attP*^*-Dl-HA* twin clones, although Dl^attP^-DlK2R-HA accumulated to higher levels in the plasma membrane than Dl^attP^-Dl-HA (Fig. 4C–E’’’, see Additional file 1: Fig. S3 for more examples). This result shows that the increased presence of Dl^attP^-DlK2R-HA in the plasma membrane correlates with reduced signalling and supports the notion that ubi-dependent endocytosis is required for normal levels of Dl-signalling and abundance in the apical membrane.

The development of the wing margin depends on continuous Notch signalling along the dorsoventral (D/V) compartment boundary [37]. The signalling results in the expression of Wg, which is crucial for margin formation and is initiated at the boundary of Fringe (Fng) expressing and non-expressing cells, by mutual signalling via Ser and Dl [38]. We found that the stripe of Wg expression along the D/V boundary was grossly normal in *Dl*^*attP*^*-DlK2R-HA*/*Df* wing discs, indicating that DlK2R-HA can provide sufficient activity for expression of Wg and margin formation (Fig. 4F–G’, arrow). This is in line with the finding that the adult flies have a normal looking margin (Fig. 2G). To test whether signalling along the D/V boundary by Dl^attP^-DlK2R-HA is weaker than by Dl^attP^-Dl-HA, we monitored the ability of the Dl^attP^ variants to activate Notch at an ectopic boundary of Fng-expressing and non-expressing cells in the wing primordium. Ectopic expression of Fng with *ptc*Gal4 induces an ectopic stripe of Wg expression in the ventral compartment of the wing anlage that straddles the anterior–posterior compartment boundary [39] (Fig. 4H, arrow). The stripe depends on mutual endogenous Dl/Ser signalling, but is initiated by Dl. Its length depends on the level of Dl signalling [39–41]. Consequently, the Fng-induced ectopic stripe is shorter in *Dl* heterozygous than wildtype wing discs [39, 41] (Fig. 4I, J, arrow). In *Dl*^*attP*^*-Dl-HA*/*Df* flies, the expression of Fng induced a stripe of ectopic Wg expression of similar length than in Dl heterozygous flies, confirming the full functionality of Dl^attP^-Dl-HA (Fig. 4J). In contrast, the ectopic stripe induced by Fng in *Dl*^*attP*^*-DlK2R-HA/Df* discs was much shorter, confirming a substantial reduction of the signalling activity of Dl^attP^-DlK2R-HA, compared to Dl^attP^-Dl-HA or endogenous Dl (Fig. 4J, K, arrow, quantification in L).

Altogether, the results extend our previously reported ones, achieved with over-expression, by showing that the loss of the Ks in the ICD causes a significant reduction in the activity of Dl in Mib1-dependent processes.

### Ubi is required for efficient trans-endocytosis of the extracellular domain of Notch during Dl signalling

**Fig. 5:**
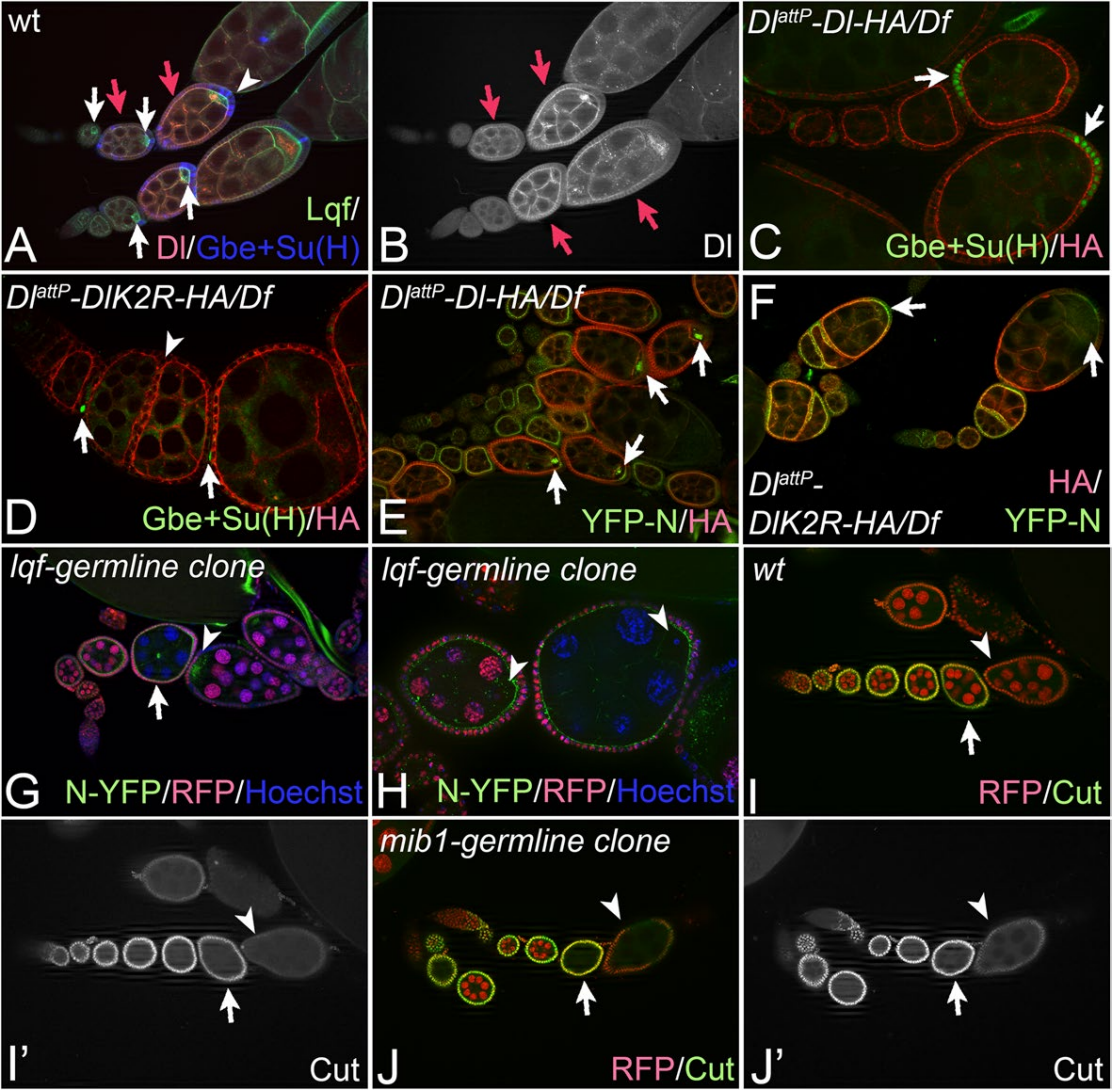
Analysis of oogenesis of *DlattP-DlK2R-HA/Df* flies. **A**, **B** Expression of Lqf-GFP, Gbe + Su(H) and Dl::mCherry in wildtype ovarioles. The red arrows point to egg chambers in stages 5–7 where Dl is up-regulated. The white arrows in **A** highlight the up-regulation of expression of Lqf/Epsin in the oocyte of maturing egg chambers. The arrowhead in **A** points to the constant expression of Gbe + Su(H) in the follicle epithelium, most prominent close to the oocyte. **C**, **D** Expression of Gbe + Su(H) in the follicle epithelium of egg chambers of *Dl*^*attP*^*-Dl-HA*/*Df* (**C**) *andDl*^*attP*^*-DlK2R-HA*/*Df* (**D**) flies. The arrows points to the expression of Gbe + Su(H), which is strongly reduced in the *Dl*^*attP*^*-DlK2R-HA*/*Df* egg chambers. Note the fusion of the egg chambers in the *DlattP-DlK2R-HA/Df* ovariole highlighted with the arrowhead in **D**. **E**, **F** Trans-endocytosis of the YFP-Notch ECD in the egg chambers of *Dl*^*attP*^*-Dl-HA*/*Df* (**E**) and *Dl*^*attP*^*-DlK2R-HA*/*Df* (**F**) ovarioles. It is best seen in the developing oocyte of egg chambers, highlighted with the arrows in (**E**). The trans-endocytosis of the ECD is dramatically reduced or absent in the *Dl*^*attP*^*-DlK2R-HA*/*Df* oocytes (**F**, arrows). **G**, **H** Clonal analysis of the function of *lqf* in the female germline. The loss of *lqf* function dramatically reduces the trans-endocytosis of YFP-NECD in the oocyte. The arrowheads point to the oocyte of the egg chambers. **H** Magnification of the egg chambers of the region highlighted with the arrow in **G**. Note that the oocyte of the younger wildtype (smaller) egg chamber already accumulated YFP-NECD, while no YFP-NECD can be detected in the *lqf*-mutant oocyte of the adjacent, older chamber. **I–J’***mib1* function is not required during signalling of Dl from the germline to the follicular epithelium. Clonal analysis of the function of *mib1* in the female germline. Expression of Cut in a wt ovariole (**I**, **I’**) and an ovariole carrying egg chambers with a *mib1* mutant germline (**J**, **J’**). **I**, **I’** Expression of Cut is down-regulated during stages 5–7 as a consequence of Dl signalling from the germline. The arrow points to an egg chamber in stage 6/7 that expresses Cut in the follicle epithelium, the arrowhead to an adjacent older egg chamber in stage 8/9 where Cut expression has been terminated because of the activation of the Notch pathway. **J**, **J’** The arrow in **J** points to an egg chamber with a *mib1* mutant germline expressing Cut, the arrowhead highlights an *mib1* egg chamber in stage 8/9, which has terminated Cut expression despite the lack of *mib1* function in the germline. This indicates that Dl can sufficiently signal to the epithelium despite the lack of *mib1* function. In contrast, the ectopic stripe induced by Fng in *Dl*^*attP*^*-DlK2R-HA/Df* discs was much shorter, confirming a substantial reduction of the signalling activity of Dl^attP^-DlK2R-HA, compared to Dl^attP^-Dl-HA or endogenous Dl (Fig. 4J, K, arrow, quantification in L)

Notch signalling initiated by Dl plays an important role in the female germline. *Dl*^*attP*^*-DlK2R-HA*/*Df* and homozygous *Dl*^*attP*^*-DlK2R-HA* females deposited only few eggs. This factual sterility is probably caused by non-penetrant defects that are characteristic for Notch loss of function, such as fused egg chambers (Fig. 5A, D, F). We therefore monitored the activity of Notch in the female germline of the *Dl*^*attP*^*-DlK2R-HA/Df*, and *Dl*^*attP*^*-Dl-HA/Df* flies during a prominent Dl-signalling event from the germline cells to the cells of the follicle epithelium that occurs during stages 5–7 of oogenesis to induce their differentiation [42, 43]. The signalling is achieved by the strong up-regulation of Dl expression during these stages in the germline cells and results in the termination of expression of Cut and the induction of expression of Gbe + Su(H) ([42–44], Fig. 5A, B, red arrows). The expression of Gbe + Su(H) is especially strong in follicle cells at the posterior pole, close to the oocyte (Fig. 5C, white arrows). The exceptional strong signalling at this site is probably induced by the observed strong expression of Epsin (encoded in *Drosophila* by *lqf*) in the oocyte (Fig. 5A, white arrows). We found that during the described stages, Gbe + Su(H) expression is strongly reduced in *Dl*^*attP*^*-DlK2R-HA/ Df*, compared to *Dl*^*attP*^*-Dl-HA/Df* flies, indicating a reduced activity of Dl^attP^-DlK2R-HA also in this process (Fig. 5C, D, arrows). The weaker signalling probably causes the observed oogenesis defects observed in *Dl*^*attP*^*-DlK2R-HA/Df* and *Dl*^*attP*^*-DlK2R-HA* homozygous flies and explains their strongly reduced fertility.

Previous work revealed that trans-endocytosis of the NECD from follicle cells into the germline, where Notch is not expressed, can be observed with exceptional clarity during the described Dl signalling event at stages 5–7 of oogenesis [42, 43]. It is best observed in the oocyte (Fig. 5E, arrows). To monitor this trans-endocytosis event in *Dl*^*attP*^*-DlK2R-HA/Df* flies, we used YFP-Notch, a functional Notch protein trap with YFP inserted in its ECD [45]. In contrast to *Dl*^*attP*^*-Dl-HA*/*Df* flies, YFP-NECD was dramatically reduced in *Dl*^*attP*^*-DlK2R-HA*/*Df* oocytes, indicating a strong reduction of trans-endocytosis of the NECD from the follicle cells (Fig. 5E, F, arrows). This is compatible with the notion that the Ks in the ICD enable Dl to exert a strong pulling force on Notch via endocytosis to efficiently activate the pathway.

In agreement with the notion that Epsin is required for the trans-endocytosis of NECD and the generation of pulling force by Dl, we found that the YFP-NECD signal was also strongly reduced in oocytes of *lqf* mutant egg chambers (Fig. 5G, H, [5–7]).

Altogether, these results indicate that ubi of Dl and its subsequent Epsin-mediated endocytosis are required for trans-endocytosis of NECD during activation of the Notch pathway in the female germline. Since trans-endocytosis of NECD is a result of successful pulling of Dl on Notch in trans and Epsin is required for the generation of a strong pulling force, the result support a requirement of ubi during pulling force generation.

Interestingly, we found that loss of *mib1* function in the germline has no detectable effect on Dl-mediated down-regulation of expression of Cut in the follicle epithelium during stages 5–7, suggesting that in this signalling event, either Neur-mediates Dl-signalling or none of the two E3-ligases is required (Fig. 5I–J’).

### Neur-induced ubi-independent signalling prevents embryonic lethality of DlattP-DlK2R flies

Previous work showed that the E3-ligase Neur plays an important role in Dl-induced Notch signalling during neurogenesis [19, 46]. Similar to the absence of Dl, absence of Neur results in embryonic lethality caused by the development of a neurogenic phenotype [19]. Our previous work showed that Neur can strongly activate Dl in a K-independent manner in over-expression experiments [27]. Combined, these findings suggest that the embryonic lethality of *Dl*^*attP*^*-DlK2R/Df* or homozygous flies is prevented by the ability of Neur to activate DlK2R via the ubi-independent pathway, also under endogenous DlK2R-expression conditions. To rigorously test this assumption, we first analysed the Neur-mediated selection of the SOP in *Dl*^*attP*^*-DlK2R/Df* wing discs*.*

The mechano-sensory bristles are a major part of the adult peripheral nervous system. Their formation depends on Neur- and Dl-mediated Notch signalling [47]. Each bristle is made of four cells, which are the progenies of the sensory organ precursor cell (SOP). Notch signalling is required to select the SOP from an equivalence group, the proneural cluster. The loss of Notch activity causes the formation of an excess of SOPs, since all proneural cluster cells adopt the SOP fate (neurogenic phenotype). We analysed the emergence of the SOP of the large bristles in the notal region of the wing imaginal discs, which arise during the third larval instar stage. To do so, we used the expression of *sca*-lacZ, which detects the whole proneural cluster, of E(spl)m8-SM-GFP, which reports only high proneural activity, and of neur-RFP, a marker for the emerging SOP (Additional file 1: Fig. S4A-A’’’) [36]. We found no obvious differences in the expression of these markers between the wildtype and the two *Dl*^*attP*^*-X/ Df* genotypes, indicating that SOP selection is grossly normal in *Dl*^*attP*^*-DlK2R-HA/ Df* flies (Additional file 1: Fig. S4A-C’’’).

To directly show that Neur is involved in SOP selection in *Dl*^*attP*^*-DlK2R-HA* flies, we depleted its function by expressing an UAS *neur-RNAi* construct throughout the notal region with *ci*Gal4 in wing discs of *Dl*^*attP*^*-DlK2R-HA*/*Df* flies. The depletion resulted in the formation of a weak neurogenic phenotype, as it is characteristic of loss of Neur function (Additional file 1: Fig. S4D, E, arrow, [36, 48]). This observation suggests that correct SOP selection in *Dl*^*attP*^*-DlK2R-HA*/*Df* flies depends on Neur.

**Fig. 6:**
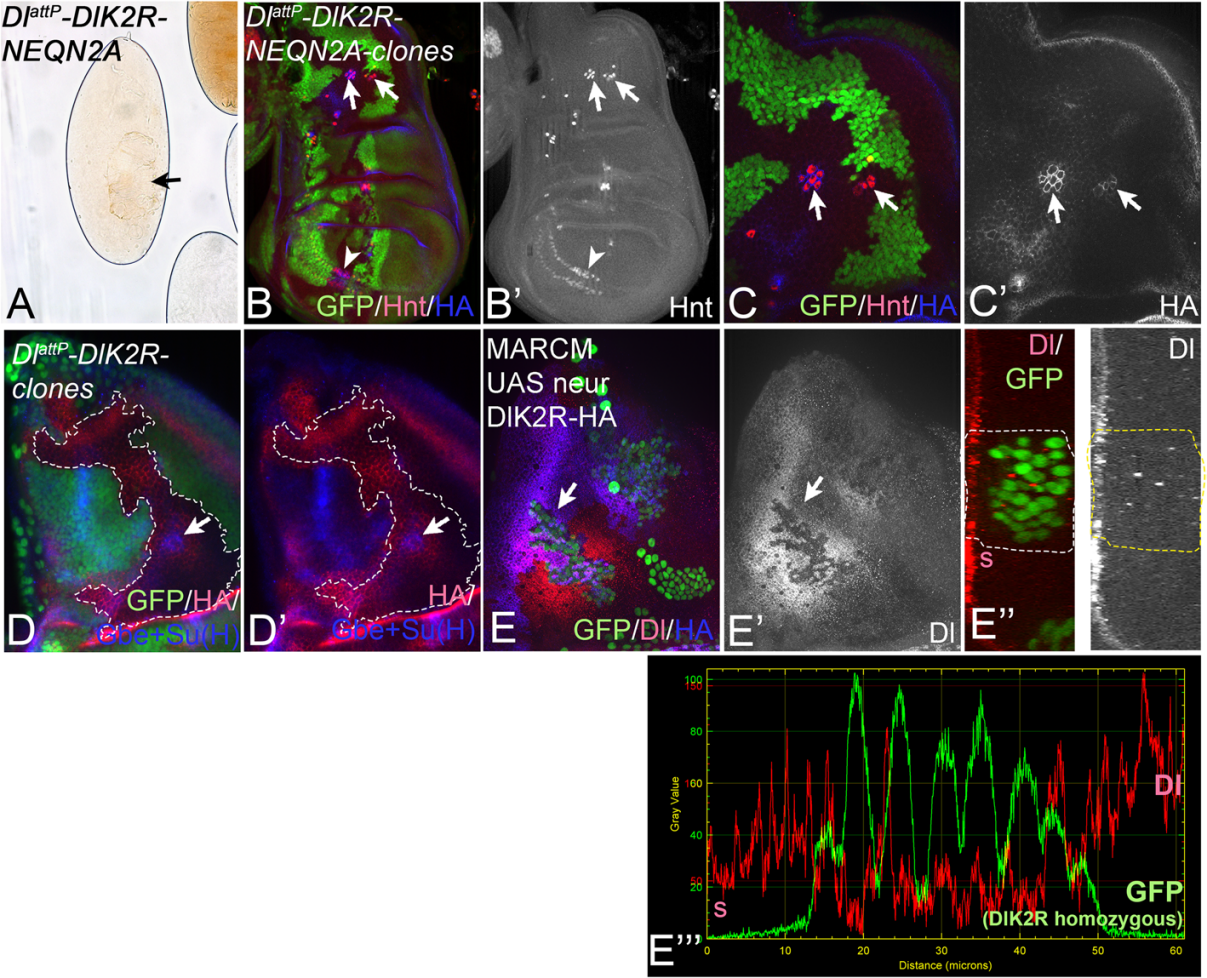
Neur-dependent Dl-signalling prevents a neurogenic phenotype of homozygous *Dl*^*attP*^*-DlK2R-HA* flies. **A**–**C** Analysis of *Dl*^*attP*^*-DlK2R-NEQN2A-HA*. **A** Cuticle preparation of a homozygous *Dl*^*attP*^*-DlK2R-NEQN2A-HA* embryo*.* It shows the severe reduction of cuticle (arrow) that is characteristic for the neurogenic phenotype. **B**–**C’** Clonal analysis of *Dl*^*attP*^*-DlK2R-NEQN2A-HA* in wing discs. Clones are labelled by the loss of GFP. Supernumerary SOPs, labelled by Hnt-expression, develop in the homozygous *Dl*^*attP*^*-DlK2R-NEQN2A-HA* clones in the notum (arrows) and the anterior wing margin (arrowhead), indicating that Dl^attP^-DlK2R-NEQN2A cannot mediate the Neur-dependent selection of the SOP. **C**, **C’** Magnification of the notal region highlighted in **A** with the arrows. It reveals that the developing SOPs accumulate DlK2R- NEQN2A-HA in their plasma membranes to high levels (arrows). **D**, **D’** A wing disc bearing large homozygous *DlattP-DlK2R-HA* clones, labelled by loss of GFP and outlined in white. The arrow points to the SOP at the prominent halo of Gbe + Su(H) expression surrounding the emerging SOP at the tr1/APA position in the homozygous clones. The halo reveals the presence of Neur-mediated signalling of the ligands. **E**–**E’’’** A wing disc bearing Neur-expressing MARCM clones which are also homozygous for *Dl*^*attP*^*-DlK2R-HA* (genotype: *hs*Flp *tub*Gal4 UAS GFPnls; + / UAS *neur*; FRT 82B *tub*Gal80/ FRT82B *Dl*^*attP*^*-DlK2R-HA*). The arrow in **E**, **E’** points to a *Dl*^*attP*^*-DlK2R-HA* homozygous clone shown at higher magnification in the z-section in **E’’**. In contrast to *Dl*^*attP*^*-DlK2R-HA* clones, the abundance of DlK2R in the plasma membrane (**E’**) is strongly reduced if Neur is additionally expressed. **E’’’** Pixel intensity measurement of the apical region including the clone shown in **E’’**. The levels of apical Dl are reduced in the GFP-positive MARCM clone (high GFP). Note, that an increase in the abundance of DlK2R-HA is normally observed in the homozygous cells (see Fig. 3A–A’’’)

To test whether Neur-dependent Dl-signalling is required also for correct embryogenesis, we mutated the Neur-binding site (NEQN) in the ICD of in *Dl*^*attP*^*-Dl-HA* and *Dl*^*attP*^*-DlK2R-HA* to alanine (NEQN2A) [17, 49]. We found that, in contrast to *Dl*^*attP*^*-Dl-HA* and *Dl*^*attP*^*-DlK2R-HA*, *Dl*^*attP*^*-Dl-NEQN2A-HA* and *Dl*^*attP*^*-DlK2R-NEQN2A-HA* homozygous flies die during embryogenesis and displayed the cuticle phenotype, which is characteristic for neurogenic mutants (Fig. 6A, Additional file 1: Fig. S4F, compare with Fig. 1B, B’). We also monitored SOP selection in wing discs bearing cell clones that are homozygous for *Dl*^*attP*^*-DlK2R-NEQN2A-HA* or *Dl*^*attP*^*-DlK2R-NEQN2A-HA* (Fig. 6B–C’, Additional file 1: Fig. S4G-G’’). We observed an excess of SOPs in the clones, confirming that Neur requires the binding to Dl to mediate ubi-independent Dl activation during also during SOP selection (Fig. 6B–C’, Additional file 1: Fig. S4G-G’’ arrows). Moreover, in discs bearing *DlattP-DlK2R-HA* clones, we observed the typical halo of Gbe + Su(H) expression in homozygous clones at certain positions of SOP development, which depends on Neur-mediated Dl-signalling (Fig. 6D–D’, arrow, [36, 50]). Altogether, these findings show that Neur is required and sufficient for the correct ubi-independent signalling of DlK2R during neurogenesis. They also highlight the necessity of direct binding of Neur to Dl for ubi-independent Notch signalling during embryonic neurogenesis to prevent the development of the neurogenic phenotype.

### Neur induces the endocytosis of DlK2R

Neur has been shown to induce the endocytosis of Dl in the emerging SOPs [15, 51]. In agreement with this notion, we found that *Dl*^*attP*^*-DlK2R-NEQN2A-HA* and *Dl*^*attP*^*-Dl-NEQN2A-HA* accumulate in the plasma membrane of the SOPs. This has been previously found for *neur* mutant SOPs and shown that it is caused by a strong reduction in endocytosis (Fig. 6C, C’, arrows, Additional file 1: Fig. S4G-G’’, arrows, [46]). Since both Dl^attP^-NEQN2A-variants are also defective in SOP selection, like the loss of function of *neur*, this finding further supports the notion that endocytosis is required for the activation of Dl by Neur. To further test whether Neur can induce also the endocytosis of DlK2R, we generated MARCM clones that express Neur in clones homozygous for *Dl*^*attP*^*-DlK2R-HA*. If Neur can induce the endocytosis of DlK2R, its observed strong accumulation in the apical plasma membrane of non-neural disc cells should be suppressed (see Fig. 3A–A’’’’, arrows). Indeed, this is what we observed. The expression of Neur leads to a strong reduction of the abundance of DlK2R in the apical membrane (Fig. 6E–E’’’). Altogether, these observations show that Neur can induce the endocytosis of DlK2R in neural and also non-neural cells, despite the lack of Ks in its ICD. They therefore confirm that Neur does not require ubi of the ICD of Dl to initiate its endocytosis and activation.

### Ubi-independent signalling of Dl depends on Lqf/Epsin

**Fig. 7:**
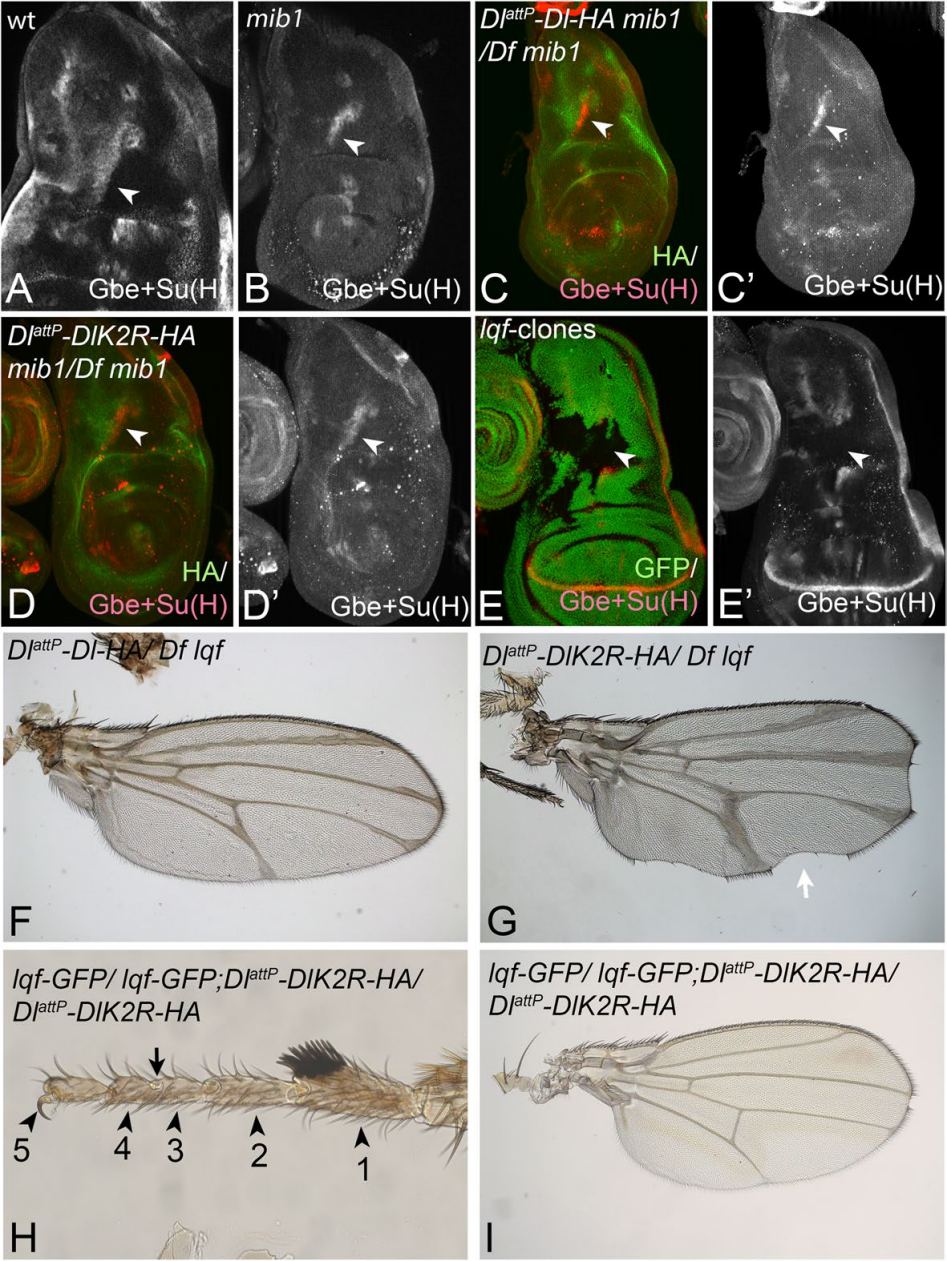
**A**–**D’** Ubi-independent Dl-signalling at the stripe3 of Gbe + Su(H) expression domain. **A** Expression of Gbe + Su(H) in a wildtype wing disc. The arrowhead points to stripe3. **B** Residual expression of stripe3 is observed in *mib1* mutant wing discs (arrowhead). **C**–**D’** Stripe3 is also present in *mib1*-mutant *Dl*^*attP*^*-Dl-HA/Df* (**C**, **C’**) and *mib1*-mutant *Dl*^*attP*^*-DlK2R-HA/Df* (**D**, **D’**) discs (arrowhead). **E,E’** A disc bearing a large *lqf-*mutant clone covering the region of stripe3 expression (arrowhead). The clone is labelled by absence of GFP. The expression of stripe3 is abolished. **F**, **G** The wing phenotype of *Dl*^*attP*^*-Dl-HA/Dfandmib1Dl*^*attP*^*-DlK2R-HA/Df* flies upon reduction of *lqf* activity. The wing of *mib1Dl*^*attP*^*-DlK2R-HA/Dflqf* flies is much more severe than that of *DlK2R-HA/Df* flies (compare with Fig. 2G)*.***H**, **I** Suppression of the phenotype of homozygous *DlattP-DlK2R-HA* flies by elevation of the copy number of *lqf*. **H** The original number of tarsal segments is re-established if two additional copies are present. The arrow points to the incomplete formation of the joint between tarsal segments 3 and 4. Note, the homozygous flies normally had only three discernible segments due to the fusion of segments 3 + 4 and 1 + 2 (see Fig. 2K). **I** The wing phenotype of homozygous *DlattP-DlK2R-HA* is strongly suppressed by elevation of the levels of Lqf (compare with Fig. 2I)

We have previously shown that over-expressed DlK2R-HA can induce weak activation of Notch in the absence of *mib1* function [27]. In this context, we found that part of stripe3 expression of Gbe + Su(H)-lacZ depends on Dl-mediated Notch signalling, but not on *mib1* function, suggesting that this part of stripe3 expression is induced by endogenous ubi-independent Dl-signalling (Fig. 7A–C’, arrowhead) [27]. To further support this notion, we monitored expression of Gbe + Su(H) in *mib1* mutants were only one copy of *Dl*^*attP*^*-DlK2R-HA* is present. The residual stripe3 expression was still present in these *mib1 Dl*^*attP*^*-DlK2R-HA*/ *mib1 Df* flies, confirming the existence of the ubi-independent signalling activity of Dl (Fig. 7C–D’, arrowhead).

We next asked whether the expression of stripe3 and therefore ubi-independent signalling of Dl requires the function of Epsin. Indeed, the clonal analysis revealed that stripe3 expression was lost completely in *lqf-*mutant cell clones, indicating the requirement of Epsin for its expression (Fig. 7E, E’, arrowhead). Moreover, we found that the phenotype of adult *Dl*^*attP*^*-DlK2R-HA/Df* flies was enhanced by removal of one copy of *lqf*. The *lqf*/ + ; *Dl*^*attP*^*-DlK2R-HA/Df* flies displayed nicks in the wing margin and enhanced broadening of the wing veins compared to *Dl*^*attP*^*-DlK2R-HA/Df* flies (Fig. 7F, G, compare with Fig. 2G). In a complementary experiment, we found that the elevation of *lqf* activity, achieved by introduction of two copies of *lqf-GFP*, a genomic construct where Lqf-GFP expression is controlled by the endogenous promoter, suppresses the mutant phenotype of homozygous *Dl*^*attP*^*-DlK2R-HA* flies ([27, 52], Fig. 7H, I, compare with Fig. 2A, K, D, I). Altogether, the results also indicate that ubi-independent Dl-signalling requires Epsin*-*mediated endocytosis. The suppression of the homozygous *Dl*^*attP*^*-DlK2R-HA* flies by increasing the levels of Lqf also suggests that Epsin is involved in the regulation of CI.

### The UIMs are not essential for Epsin-mediated activation of Dl

**Fig. 8:**
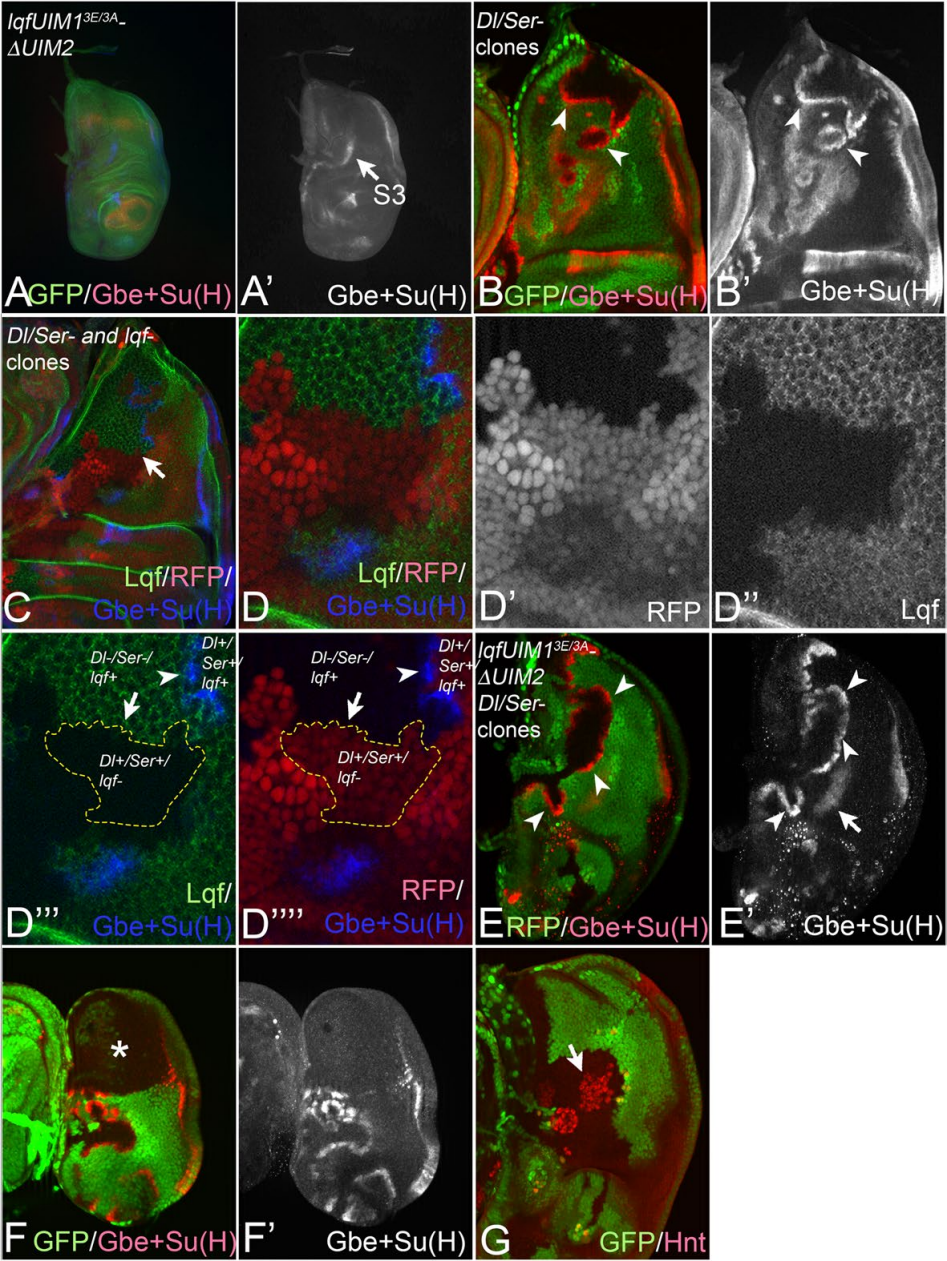
RCI in *LqfUIM1*^*3E/3A*^*-∆UIM2-GFP* wing discs. **A**, **A’** Gbe + Su(H) expression in *LqfUIM1*^*3E/3A*^*-∆UIM2-GFP* wing discs. The arrow points to the residual expression of stripe3. Compare with Fig. 7A, B. **B**, **B’** Detection of RCI in the notal area of wing discs by induction of *Dl/Ser*-double-mutant clones. Ectopic expression of Gbe + Su(H) is activated in mutant cells at the clone boundary (arrowheads). **C–D’’’’** A wing disc bearing lqf- and Dl/Ser mutant clones. The analysed disc is *lqf*-mutant, rescued by a GFP-tagged *lqf* construct, expressed under control of the endogenous *lqf*-promoter. In the rescued discs, the induced *Dl/Ser* double-mutant clones are labelled by the absence of RFP. The *lqf*-mutant clones are labelled by absence of the GFP signal caused by the loss of Lqf-GFP. The activity of Notch is revealed by the expression of Gbe + Su(H). The arrowheads highlight regions with strong ectopic induction of Notch activity (Gbe + Su(H) expression) in *Dl/Ser*-mutant cells at the clone boundary. **B–B’’’’’** Independent induction of *lqf-* and *Dl/Ser*-clones in discs generates regions devoid of *lqf* functions adjacent to *Dl/Ser*-mutant *lqf* + clones. The clones are outlined in yellow in **D’’’**, **D’’’’**. The expected induction of Notch activity (Gbe + Su(H) expression) upon RCI is absent (arrow), indicating that the Dl/Ser expressing cells require the function of *lqf*. The arrowhead points to a lqf + region adjacent to a *Dl/Ser*-mutant clone. As expected the *Dl/Ser*-mutant cells initiate ectopic expression of Gbe + Su(H). The experiment confirms that Lqf is required in the signalling cells to induce Notch activity in the mutant boundary cells. **E–F’***Dl/Ser*-clones in *LqfUIM1*^*3E/3A*^*-∆UIM2-GFP* wing discs. **E**, **E’** Gbe + Su(H) is also activated in the *Dl/Ser*-double-mutant boundary cells, indicating RCI occurs in cells with a Lqf variant without functional UIMs (arrowheads). Thus, *Dl/Ser* can signal despite the absence of functional UIMs in Lqf. **F**, **F’** As expected, no induction of Notch activity is observed if the *Dl/Ser*- mutant clone incudes the whole notum, because no boundary of ligand-expressing and non-expressing-cells are present (asterisk in (**F**)). **G** The loss of *Dl/Ser*-function in *LqfUIM1*^*3E/3A*^*-∆UIM2-GFP* wing discs results in a strong neurogenic phenotype, confirming that the ligands are required for SOP selection in this genetic background

Epsin has two UIMs which are thought to be essential for the interaction with Dl. Since Epsin is involved in all modes of Dl signalling, including ubi-independent signalling, we wondered whether Epsin absolutely requires its ubiquitin-binding UIMs to activate Dl. We have previously shown that *lqf* (*epsin*) null mutant flies, which die during embryogenesis, can be partially rescued by the presence of a Lqf/Epsin variant that lacks functional UIMs (LqfUIM1^3E/3A^-∆UIM2-GFP) and therefore cannot interact with ubiquitylated cargo [27, 52]. LqfUIM1^3E/3A^-∆UIM2-GFP is endogenously expressed under control of the *lqf* promoter (*LqfUIM1*^*3E/3A*^*-∆UIM2-GFP* flies). *LqfUIM1*^*3E/3A*^*-∆UIM2-GFP* flies developed to the pharate adult state and display strong defects that resembled that of *mib1* mutants [27, 52]. In the wing disc of these *LqfUIM1*^*3E/3A*^*-∆UIM2-GFP* flies, the same residual stripe3 expression as in *mib1* mutants was observed, suggesting that ubi-independent Dl/Ser-signalling takes place and does not require the UIM in Epsin (Fig. 8A, A’, arrow, compare with Fig. 7A, B). To rigorously assay Dl-signalling at the endogenous level in the discs of *LqfUIM1*^*3E/3A*^*-∆UIM2-GFP* flies, we exploited the phenomenon of relieve of CI (RCI). In wildtype wing discs, the presence of CI can be revealed by the induction of cell clones lacking *Dl* and *Ser* function [27, 29, 53]. At the boundary of these clones, the adjacent wildtype Dl/Ser-expressing cells signal to the mutant boundary cells, since Notch in the mutant cells is released from the cis-inhibitory interaction by the removal of the ligands. The activation of Notch in the mutant cells at the clone boundary can be revealed by monitoring the expression of Gbe + Su(H) (Fig. 8B, B’, arrowheads). We first confirmed that the ligands of the wt cells require the function of *lqf* to signal to the mutant boundary cells at the clone boundary ([10], Fig. 8C–D’’’’). Thus, Epsin/Lqf is essential for Dl signalling also during RCI.

When we induced *Dl/Ser*-double mutant cell clones in *LqfUIM1*^*3E/3A*^*-∆UIM2-GFP* discs, we also observed strong activation of expression of Gbe + Su(H) in the *Dl/Ser*-mutant boundary cells (Fig. 8E–F’). This indicates that RCI is present in the partially rescued *lqf* mutant discs, despite the fact that LqfUIM1^3E/3A^-∆UIM2-GFP cannot interact with Dl and therefore cannot exert its adapter function. Since the control experiment showed that Epsin is essential for Dl signalling during RCI, the result suggests that Epsin must not necessarily directly bind ubiquitylated Dl to induce a certain level of Dl activity or interacts in a so far not appreciated way. In addition, the results are also compatible with a role of Lqf/Epsin during CI.

Note, that the loss of *Dl/Ser* function in the partially rescued discs results in a strong neurogenic phenotype, indicating also that ubi-independent Lqf-mediated SOP selection requires Dl/Ser-signalling (Fig. 8G, arrow). This finding is also compatible with the notion that DlK2R-mediated ubi-independent selection of the SOP depends on Lqf/Epsin.

### Gut homeostasis in DlattP-DlK2R-HA flies

**Fig. 9:**
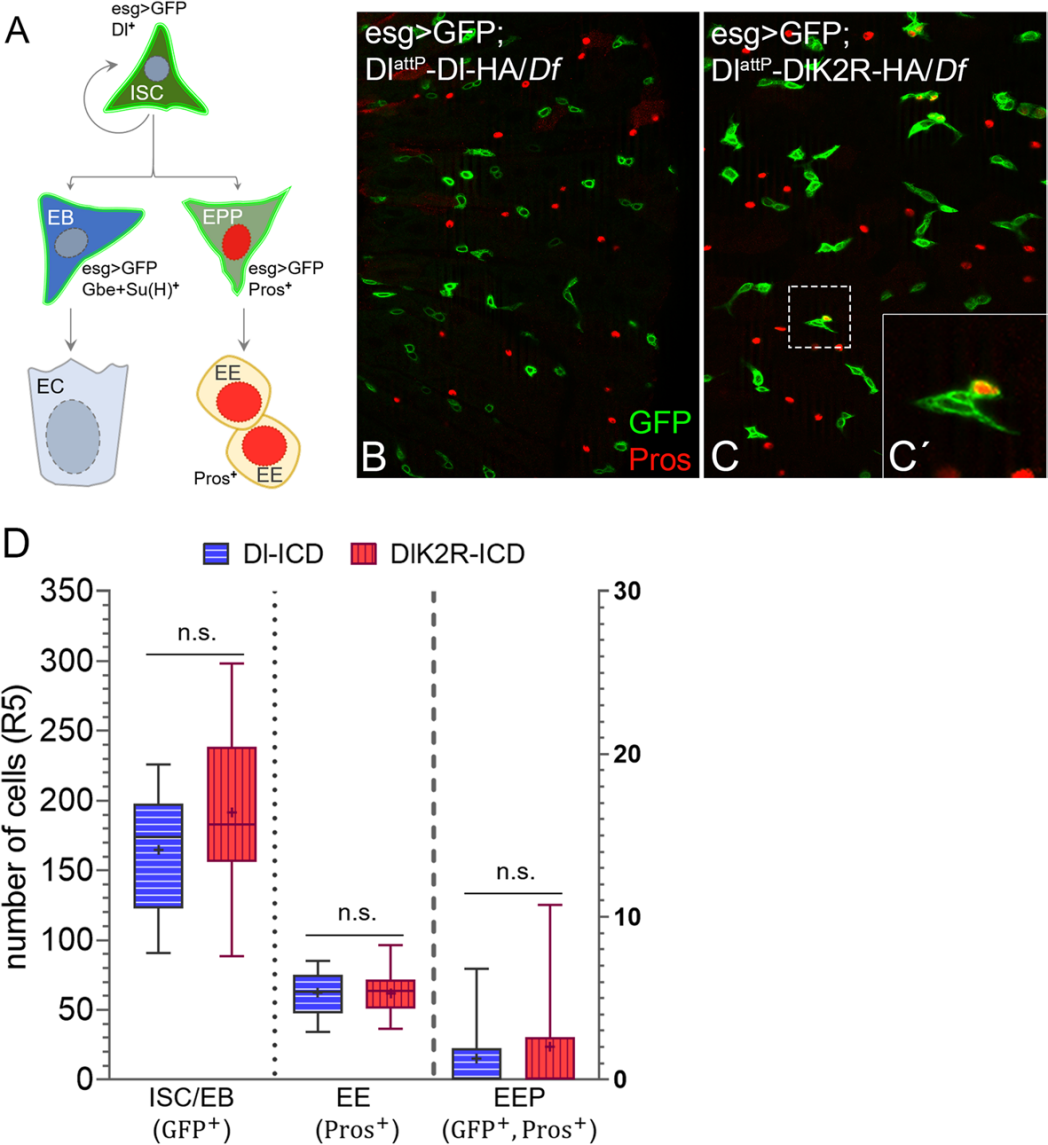
Gut homeostasis in D^lattP^-DlK2R-HA/Df flies. **A** Schematic of the ISC lineage, giving rise to the EC and EE cells. **B–C’** A representative picture of the R5 region of the gut of *Dl*^*attP*^*-DlK2R-HA/Df* and *D*^*lattP*^*-Dl-HA/Df* flies. The expression of Pros and Esg-GFP are shown. The insert **C’** shows the rare Pros and Esg-GFP double positive EEP precursor that differentiate into EEs. **D** Quantification of the number of ISCs, EEs and EEPs in the two genotypes revealed no significant differences (*n* = 11, 15). Error bars are standard error of the mean (SEM). A two-tailed student´s *t*-test was used for statistical analysis (*p < 0.05, **p < 0.01; ***p < 0,001)

Dl is required for the homeostasis of the gut epithelium of the imago. This epithelium consists of five cell types, the intestinal stem cell (ISC), which divides asymmetrically to give rise either to the enteroblast (EB) or an entero-endocrine precursor (EEP) (Fig. 9A). The EB differentiates into the endocrine cell (EC), the EEP into the entero-endocrine cells (EE) [54]. The EB and EEP fate requires Dl signalling from the adjacent ISC. High signalling induces the EB fate in the arising precursor cell, whereas low signalling induces the EEP fate.

To monitor gut homeostasis in our *Dl*^*attP*^*-*variants, we used the expression of Escargot (Esg) to label the ISCs and EBs and that of Prospero (Pros) to label the EEs [54]. The rare EEPs can be discriminated from the other cell types by its double labelling with Pros and Esg (Fig. 9B, C’). We did not find any significant difference in the number and distribution of these markers between the two Dl^attP^ variants, indicating that homeostasis of the gut epithelium is not grossly affected by the loss of ubi of Dl (Fig. 9B–D). Thus, ubi appears to be dispensable for Dl signalling during homeostasis of the gut epithelium. Note, that Dl signalling during gut homeostasis depends on Neur [54]. Thus, it appears that Neur can sufficiently activate Dl in a ubi-independent manner also in this process.

## Discussion

Here, we present the results of our continued investigation of the meaning of ubi for the function of the DSL ligand Dl during Notch signalling. Previous investigations were performed under conditions of over-expression, either in the wing imaginal discs of *Drosophila* or in mammalian cell culture [27, 55]. This work did cover only a small number of developmental processes and focussed primarily on aspects of signalling. Thus, it lacked the organismal perspective. For our analysis, we generated knock-in alleles *Dl*^*attP*^*-DlK2R-HA*, a variant with the Ks of its ICD replaced by Rs and *Dl*^*attP*^*-Dl-HA* as a control. It was previously shown that DlK2R cannot be ubiquitylated by Mib1 and Neur in S2 cells [27]. We found that the sole presence of one copy of *Dl*^*attP*^*-DlK2R-HA* in the genome of *Drosophila* provided sufficient Dl activity to complete development. Thus, the previously detected ubi-independent activity of DlK2R-HA observed upon over-expression is not an artefact, but a component of the normal Dl signal, which is surprisingly potent. Despite this surprise, the analysis also revealed that Dl^attp^-DlK2R-HA is significantly less active than Dl^attP^-Dl-HA in several processes, indicating that, while not absolutely essential, the Ks and their ubi are required for the full activity of Dl and robustness of the signal. Importantly, the processes affected by the loss of the Ks are mainly the non-neural Dl-regulated processes, which are mediated by Mib1, indicating that Mib1 operates largely via ubi and lacks the ubi-independent activation mode of Neur.

*mib1* mutant flies die as pharate adults, which display a severe Notch loss of function phenotype in non-neural processes, such as the wing, eye, head and leg development [14]. The severity of the phenotype indicates that the ubi of the ligands is an important requirement for Notch signalling in these processes. Thus, why is the phenotype of *Dl*^*attP*^*-DlK2R-HA* flies so much weaker than that of *mib1* mutants? One obvious reason is that there is still a considerable amount of Notch activity generated by the presence of the other ligand Ser, which is expressed in domains that largely overlap with that of Dl. Ser can be ubiquitylated by Mib1 in *Dl*^*attP*^*-DlK2R-HA/Df* (or homozygous) flies. It provides significant Notch activity in several, Mib1-dependent processes, such as wing development, where it has been shown to be the more important ligand [36, 56]. Another important factor is the residual ubi-independent activity of Dl, which we confirmed here for endogenously expressed DlK2R-HA. The combination of the two sources of ligand activity appear to be sufficient for the Mib1-dependent developmental processes to run with approximately sufficient fidelity in *Dl*^*attP*^*-DlK2R-HA* flies.

However, Ser and Mib1 are not essential for neural development in *Drosophila*, which mainly depends on Dl and Neur. The death of *Dl-* and *neur*-mutant flies during embryogenesis is caused by the development of a strong neurogenic phenotype. We here show that the observed correct execution of neural development in *Dl*^*attP*^*-DlK2R-HA/ Df* flies is explained by the ability of Neur to activate Dl independently of ubi. We show that the selection of the SOP in the wing disc fails in *Dl*^*attP*^*-DlK2R-HA* flies, if the function of Neur is depleted. Moreover, we here show that for Dl^attP^-DlK2R-HA or Dl^attP^-Dl-HA to prevent a neurogenic phenotype, it requires a functional NBM in its ICD. This confirms that Neur must be able to physically interact with DlK2R for its activation and suppression of the neurogenic phenotype. Our finding that, in contrast to Mib1, Neur can induce the endocytosis of DlK2R-HA, suggesting that the activation of DlK2R by Neur is achieved by an endocytosis event. Together with Epsin, this probably creates the necessary pulling force. Our results are in agreement with the previous over-expression analysis that revealed that Neur can activate DlK2R-HA as good as Dl-HA, suggesting that ubi is not important for activation of Dl by Neur [27].

Altogether, these findings suggest that the complete development of the *Dl*^*attP*^*-DlK2R-HA* flies is possible because of the activation of the Notch pathway via the combination of Ser signalling, the ubi- and E3-ligase-independent activity of Dl and its strong ubi-independent activation by Neur. The results, also indicate that non-neural, Mib1/Dl-dependent processes, such as leg and wing development, appear to require ubi of Dl by Mib1. In these processes, ubi is required for the generation of a strong Dl-signal. An interesting question for the future is whether the rules discovered for Dl apply also to the other ligand Ser.

Our results provide further evidence for the importance of endocytosis for the activity of Dl. Its importance has been previously suggested, based on the requirement of the E3 ligases. Recent work supports the meaning of endocytosis, especially mediated by Epsin, to create a tensile force that changes the conformation of Notch, which in turn enables the proteolytic release of NICD. It also provides further compelling evidence for the pulling force model for activation of Notch where the trans-endocytosis of NECD is a visible consequence [4–8]. In our analysis of oogenesis, we observed a strong reduction of the trans-endocytosis of NECD into the signal-sending germline cells in the case of *Dl*^*attP*^*-DlK2R-HA*/*Df* flies. We observed similar reduction in trans-endocytosis upon loss of *lqf/epsin* function, which has been implicated as essential for pulling force generation. Combined, these findings suggest that DlK2R generates less pulling force compared to Dl. We here also observed that DlK2R-HA accumulated to significantly higher levels in the plasma membrane than Dl-HA or endogenous Dl. In addition, less DlK2R-HA was detected in *Dmon1* mutant enlarged endosomes and that DlK2R-signalling depends on the endocytic adapter Epsin. Moreover, we showed that the combined loss of function of the two major recycling pathways did not affect the subcellular distribution of DlK2R, indicating that recycling is probably not involved in the trafficking of DlK2R and Dl. Altogether, these observations suggest that DlK2R-HA is less efficiently endocytosed, which leads to less trans-endocytosis of NECD and therefore to a reduced pulling force. It is likely that the reduced pulling force is the cause of the of the observed reduced signalling ability of DlK2R.

We were surprised in which clarity we could detect the defect in endocytosis of DlK2R-HA, as it was stronger than the loss of *mib1/lqf* function. Hence, our results raise the possibility of involvement of an additional E3 ligase in the endocytosis of Dl. This E3 ligase is probably not relevant for signalling and might induce bulk endocytosis of Dl. It is probably its irrelevance for signalling why its function in Dl endocytosis has not been recognised.

It is formally possible that the addition of the HA tag to the ICD might change the behaviour of DlK2R-HA and leads to the stronger accumulation in the plasma membrane than expected from the *mib1* mutant phenotype. However, the direct comparison of the subcellular localisation of Dl^attP^-Dl-HA with endogenous Dl using clonal analysis (Fig. 1E, E’) revealed no obvious difference in the subcellular localisation. Therefore, it is unlikely that the HA tag influences the endocytosis and endosomal trafficking. It is possible that the K2R exchange although conservative could lead conformational changes of the ICD of Dl. However, we ran the sequence through several programmes and found no predicted domain in essence; the ICD seems to be intrinsically disordered. This is also predicted by AlphaFold 2. Also theoretically possible is that the K2R exchange might create a binding site for an alternative endocytic adapter that channels DlK2R into endocytosis and therefore accounts for the residual Dl activity observed for DlK2R. However, our sequence analysis of the ICD of DlK2R did not reveal any binding site for known endocytic adapters. In addition, we have here and previously identified stripe 3 expression of Gbe + Su(H) reporting ubi-independent signalling by endogenous unmodified Dl [27]. Its expression depends on Notch, Epsin and the ligands, but not on *mib1* or the UIMs of Epsin. Moreover, we here observe RCI in *lqfUIM1*^*3E/3A*^*-∆UIM2-GFP* discs, indicating that the ligands can signal, although the encoded Epsin variant is unable to bind ubiquitylated cargo. Finally, we previously also showed that RCI exists in *mib1* mutant discs [27]. Thus, endogenous ubi-independent Dl-signalling is present during normal signalling events and is probably not a special property introduced by the K2R exchange in Dl.

Previous work has already highlighted the importance of the endocytic adapter Epsin for the activation of Notch by its ligands [5–7, 10]. We here confirm and extend this conclusion by showing that all three discovered modes of Dl signalling, the ubi- and Mib1-dependent, the ubi-independent and the Neur-dependent mode require the function of Epsin. It is worth highlighting that Neur-mediated DlK2R signalling appears to depend on Epsin. This conclusion is based on the previously made observation that Neur-dependent neurogenesis depends on *lqf*, but is nearly normal in *lqfUIM1*^*3E/3A*^*-∆UIM2-GFP* flies [27]. It is further based on our findings here. We show that the NBM in DlK2R is required for its Neur-mediated activation and that SOP selection in *lqfUIM1*^*3E/3A*^*-∆UIM2-GFP* flies depends on the presence of the ligands. What is the role of Neur and Epsin in the ubi-independent, Neur-dependent of Dl signalling mode? A possibility which we have recently proposed is that Neur could act as a co-adapter that might bind to Dl and, after auto-ubi, also to Epsin [3].

Since Epsin is required also for the two other Neur-independent modes of Dl signalling, it must also act via other mechanisms. In the Mib1-dependent mode, which occurs via ubi, Epsin probably acts via its UIMs to bind to ubiquitylated Dl, as previously suggested [3, 6, 10]. In the ubi-, Mib1- and Neur-independent signalling mode of Dl, e.g. revealed by the residual expression of stripe3, the mechanism of Epsin must be different. Previous work in *Drosophila* and mammalian cell culture suggests that Epsin is crucial for pulling force generation by the ligands [5, 6]. Our findings that trans-endocytosis of NECD is strongly impaired in *DlattP-DlK2R-HA*, or cells that have lost the function of *lqf*, further support this notion. How Epsin generates the strong pulling force is not clear. However, in contrast to the general endocytic adapter AP-2, which is not required for Notch signalling [6, 57], Epsin has two endocytic functions in addition to the recruitment of cargo (adapter function) into the endocytic pit (summarised in [3]). It additionally induces membrane curvature via its ENTH domain and organises Actin at the endocytic pit into a functional network [3, 58]. These two functions have been shown to be important for the function of Epsin during Notch-signalling and are probably crucial for generation of the pulling force and the domains mediating this function [3, 52]. In principle, these functions can be provided by Epsin without physically interacting with Dl, as long as it is present in the endocytic pit together with Dl. In the case of the Mib1-, Neur- and ubi-independent Dl signalling, it is possible that the amount of Dl that enters the endocytic pits is strongly reduced, since cargo selection by Epsin does not occur. However, it is likely that small fraction of Dl enters the endocytic pits by accident or other mechanisms. If Epsin is also present and performs the two additional force generating functions, this small fraction of Dl could create a reduced but significant force. This model is compatible with our finding that elevation of the levels of Epsin achieved by introduction of two extra copies of *lqf* strongly suppresses the phenotype of homozygous *Dl*^*attP*^*-DlK2R-HA* flies, since it enhances the chance of the presence of Lqf and/or also its concentration in the endocytic pit. It is also strongly supported by our finding that Dl can signal during RCI in *lqf* mutant discs rescued by LqfUIM1^3E/3A^-∆UIM2-GFP, but cannot signal in cells that lack the complete function of *lqf*.

It is worth mentioning here that an elegant recent study has shown that in the nematode *C. elegans*, Epsin-mediated endocytosis is not essential for ligand-induced Notch signalling. It shows the *C. elegans* Notch-orthologs LIN-12 and GLP-1 require less tensile forces for activation because of the lack of a hydrophobic leucine plug in their negative regulatory regions (NRRs), which are the force sensing units of Notch receptors [7]. Replacing the NRR of Notch by that of LIN-12 generates a receptor that can be efficiently activated by DlK2R in *Drosophila*. Thus, in principle, Notch can be activated by its ligands without ubi and endocytosis. Our analysis provides more evidence for this notion.

We here provide evidence that Dl^attP^-DlK2R-HA has increased cis-inhibitory abilities. This is indicated by the observation that (1) the increase of Dl^attP^-DlK2R-HA to two copies in the genome severs the mutant phenotype, instead of abolishing it and (2) the suppression of the phenotype caused by two copies of *Dl*^*attP*^*-DlK2R-HA* by a concomitant increased the levels of Notch. We found that DlK2R is less endocytosed and accumulates in the plasma membrane. Previous work showed that the over-expression of Dl leads to more CI [24, 39, 59] Thus, it appears that the higher abundance of DlK2R in the plasma membrane is responsible for the increase in CI. The finding that elevation of the levels of Epsin suppresses the phenotype of homozygous *Dl*^*attP*^*-DlK2R-HA* flies in a similar manner as Notch, combined with the existence of RCI in *LqfUIM1*^*3E/3A*^*-∆UIM2-GFP* discs, indicates that Epsin is also involved in the regulation of CI. The determination of the precise role of Epsin in the regulation of CI should be explored in the future. We previously showed that CI is increased in *mib1* mutants, supporting a crucial role of endocytosis in the suppression of CI [27].

Altogether, our findings therefore support a model where endocytosis is at the heart of the properties of Dl. Its reduction, either by loss of the ability to get ubiquitylated (e.g. DlK2R), or by loss of the activity of E3-ligases, increases the levels of Dl at the plasma membrane. This leads to an increase of cis-inhibitory interactions with Notch and, consequently, to less available Notch molecules to engage in transactivation.

## Conclusions

Our analysis reveals that the ubi of the ICD is not essential for activation of the Notch pathway by Dl. One main reason is the ubiquitylation-independent activation of Dl by the E3 ligase Neur. Thus, Neur can act in a mode different from the other E3 ligase involved in Notch signalling, Mib1. It is likely that, in this second mode, Neur acts as an endocytic co-adapter together with Epsin to induce Dl-mediated Notch activation. Endocytosis, either ubi-dependent or ubi-independent is required for the activation of Notch by Dl and regulation of CI.

## Methods

### Fly strains

*Dl*^*attP*^ [28], *Dl*^*attP*^*-Dl-HA* FRT82B, *Dl*^*attP*^*-Dl-K2R-HA* FRT82B and *Dl*^*attP*^*-Dl-NEQN2A-HA* FRT82B, *Dl*^*attP*^*-Dl-K2R-NEQN2A-HA* FRT82B (this study), Dl:GFP [29], *mib1*^*EY09870*^ [12, 14], Gbe + Su(H)-lacZ [60], Gbe + Su(H)-GFP [61]*, sca*-lacZ (Bloomington stock center, BSC5403), *neur*-H2B-mRFP [62], *lqfUIM1*^*3E/3A*^*-∆UIM2-GFP* [27, 52], *ptc*GAL4 [63], *ci*GAL4 [64], sna1.7GAL4 [65], UASFlp,(BSC4539), UAS neur-RNAi (VDRC10662), FRT82B (BSC2035), FRT82B ubiRFPnls (BSC30555), FRT82B ubiGFPnls (BSC32655), *Dl*^*rev10*^ e FRT82B [66], Df(3R) BSC850 (BSC27922), Df(3R) BX12 (BSC3012), E(spl)m8SM-GFP [67], *lqf*^*ARI*^ [68], *Dl*^*rev10*^ e *Ser*^*RX82*^ FRT82B [66], NiGFP [31], *Dmon1*^*mut4*^ [33], YFP-N (CPTI line, [45]), UAS *fng* [39], *lqf*-*GFP* inserted into attP22a (this study), esg-lexA, AOP-CD8::GFP (gift from T. Reiff). tubP. mCherry-Rab4 [33], UAS Rab4 RNAi (VDRC24672), UASRab11RNAi (VDRC22198), ubxFlp (BDSC 42718), *lqf*^*L71*^* FRT82B Dl*^*rev10*^* e Ser*^*RX82*^, *lqf*^*1227*^* FRT82B RFPnls* [27].

All fly experiments were carried out at 25 °C unless indicated otherwise. In experiments using tubGAL80ts, flies were kept at the permissive temperature (18 °C) and then shifted to the restrictive temperature (29 °C) for the indicated time.

### Clonal analysis

Clones were induced utilising *sna1.7*-GAL4 driving UAS-*Flp*, which induces the clones specifically in early wing and haltere imaginal discs. For *Dmon1* mutant clones, ubxFlp was used. Clones in the female germline were induced with hsFlp as described in [42].

### Antibody staining and imaging

Antibody staining was performed according to standard protocols [69] and [70]. The staining on non-permeabilised discs to detect surface Dl was performed according to [14]. Staining of *Drosophila* ovaries was performed as described in [42]

 Antibodies used:

**Table:** 

Antibody list		
Antibody (from species)	Source or Reference	Dilution
Anti HA (rat)	Roche Clone 3F10	1:500
Anti HA (Rabbit)	Cell Signaling C29F4	1:1600
Anti Dl (mouse)	DSHB C594.9B	1:500
Anti Dl (mouse)	DSHB C594.9B	1:100 (surface staining)
Anti N(extra) (mouse)	DSHB C458.2H	1:100
Anti Wg (mouse)	DSHB 4D4	1:250
Anti ß-Gal (rabbbit)	Cappel	1:1500
Anti ß-Gal (Rabbit)	Cell Signaling A257	1:1500
Anti ß-Gal (mouse)	DSHB 40-1a	1:250
Anti Rab11 (Rabbit)	Tanaka & Nakamura 2008	1:8000
Anti Cut (mouse)	DSHB 2b10	1:100
Anti Prospero (mouse)	DSHB MR1A	1:100
Anti Hnt (mouse)	DSHB 1G9	1:80
Anti Rabbit Alexa 488 (goat)	Invitrogen/Molecular Probes	1:500
Anti Rabbit Alexa 568(goat)	Invitrogen/Molecular Probes	1:500
Anti Rabbit Alexa 647 (goat)	Invitrogen/Molecular Probes	1:500
Anti Mouse Alexa 488 (goat)	Invitrogen/Molecular Probes	1:500
Anti Mouse Alexa 568 (goat)	Invitrogen/Molecular Probes	1:500
Anti Mouse Alexa 647 (goat)	Invitrogen/Molecular Probes	1:500
Anti Rat Alexa 488 (goat)	Invitrogen/Molecular Probes	1:500
Anti Rat Alexa 568 (goat)	Invitrogen/Molecular Probes	1:500
Anti Rat Alexa 647 (goat)	Invitrogen/Molecular Probes	1:500

Alexa-Fluorochrome-conjugated secondary antibodies were purchased from Invitrogen/Molecular Probes. Images were acquired with a Zeiss AxioImager Z1 Microscope equipped with a Zeiss Apotome.2.

Adult wings, legs and embryos were mounted in Hoyers Medium and Images were acquired using a Zeiss Axiophot equipped with a Zeiss MRC digital camera.

Adult flies were imaged with a Leica MZFLIII equipped with a Zeiss MRC digital camera.

Image analysis was performed using Fiji(ImageJ).

For the analysis of endosomes in *Dmon1* mutant clones, the association of the anti HA-signal with the anti Nextra signal was analysed by thresholding the Nextra signal using Fiji(ImageJ) thresholding tool. To obtain regions of interest (ROI) encompassing the accumulations of Notch in endosomes, particle analysis was performed. The ROI sets were measured, and the median fluorescence of anti HA was determined for each ROI (endosome). Three discs of each genotype, were analysed (Dl-HA and DlK2R-HA). The total number of vesicles analysed for each genotype was *n* = 1043 and *n* = 983, respectively. The obtained data were statistically analysed using Prism Graphpad 7. The comparison of Gbe + Su(H)-lacZ, anti HA and GFP fluorescence intensities in Dl^attP^Dl-HA (+ ubiGFP) and Dl^attP^-Dl-K2R-HA mosaic discs was done by drawing a ROI and subsequently analysing the fluorescence using Fiji (ImageJ) plot profile tool.

### Cuticle preparations

Cuticle of homozygous embryos were prepared according to Van der Meer (1977) [71].

### Cloning

The constructs Dl-HA and Dl-K2R-HA were seamlessly cloned into the original vector pGE-attB-Dl::EGFP via replacement of the ICD::GFP part by Gibson Assembly [72]. An additional XbaI restriction site exactly after the stop codon was introduced for the generation of further constructs. The constructs Dl-NEQN2A-HA and Dl-K2R-NEQN2A-HA were cloned by replacing a BstEII/XbaI fragment from a previous pUAST-attB-Dl-NEQN-HA/pUAST-attB-Dl-K2R-NEQN2A-HA construct [27] into pGE-Dl-HA + XbaI. The constructs were inserted into the *Dl*^*attP*^-landing site as described in [73].

Primer used: Dl_ICD_attP _for: GCA AGC AGT GCG ATG AGG AGT CCT ACG ATT C GGT GAC CTT CGA TGC CCA CC.

Dl_ICD_attP _rev: TTC CCA GGA GCC CTT CCG GAT TTT TGG AG TTA AGC GTA GTC TGG AAC GTC.

NdeI_HA_XbaI_3'UTR of Dl_for: GCACTCCGCATATGGCCTACCCATACGACGTTCCAGACTACGCTTAATCTAGAAACTCCAAAAATCCGG.

3'UTR of Dl_NheI-rev: GTTTACACATACGCGTTGGCTACGGCTAGCATTAATATTTAC.

The *lqf-GFP* rescue construct [52] was obtained from the Fischer lab and subcloned into the attB vector for insertion at the genomic site 22A.

### Supplementary Information

**Additional file 1: Fig. S1.** Expression of DlattP-constructs in various tissues compared to Dl::GFP. (A-A’’, C-C’’, F-F’’, H-H’’) Expression in various imaginal discs of the late third instar stage. (A-A’’, F-F’’) Expression in the eye imaginal disc. (C-C’’, H-H’’) Expression in the haltere, wing and leg disc.(B-B’’, G-G’’) Expression in the larval brain of the late third instar stage. The arrowhead highlights expression in the optic lobes. (D-D’’, I-I’’) Expression in stage 11 embryos. (E-E’’, J-J’’) Expression in the adult gut. Expression is largely restricted to escargot (esg-GFP) positive ISCs (E’’, J’’). **Fig. S2.** (A-B’’) Comparison of expression of Dl^attP^-Dl-HA and Dl^attP^-DlK2R-HA on the cell surface. Clonal analysis was used to twin clone that express either of the Dl^attP^-variants in homozygosity, as described in (A). (B, B’) An example of a disc bearing the clones. The arrowhead points to a Dl-HA homozygous clone. The abundance of Dl is much reduced in the cells of Dl-HA homozygous in comparison to cells of DlK2R homozygous or heterozygous clones. (B’’) Pixel density measurements of the channels in the apical membrane region highlighted by the thin line in (B, B’). It revealed that Dl-HA is significantly less abundant than DlK2R-HA in the membrane. (C-C’’’) Co-depletion of Rab4 and Rab11 by expression of RNAi constructs for 46h using a combination of ciGal4 and *tub*Gal80^ts^. It efficiently suppresses the expression of a mCherry-Rab4 construct (C’), as well as Rab11 (C’’). (D, D’) Co-depletion of Rab4 and Rab11 had no effect on the expression of DlK2R-HA, suggesting that endosomal recycling is not involved in the increase of it abundance in the membrane. **Fig. S3.** Three additional examples of wing discs with twin clones homozygous for *Dl*^*attP*^*-DlK2R-HA* or *Dl*^*attP*^*-Dl-HA* crossing the region of stripe2 at different points. **Fig. S4.** (A-C’’’) Analysis of SOP formation in *Dl*^*attP*^*-DlK2R-HA/Df* (B-B’’’) and *Dl*^*attP*^*-Dl-HA/Df* (C-C’’’) wing discs. (A-A’’’) The expression of the used marker in the wildtype is shown for comparison. (B-C’’’) No significant differences in the expression of the markers are detected, indicating that the SOP selection occurs normally in *Dl*^*attP*^*-DlK2R-HA/Df* flies. (D, E) Depletion of *neur* function by *neur*-RNAi with *ci*Gal4 in *Dl*^*attP*^*-DlK2R-HA/Df* flies causes the development of a neurogenic phenotype (arrow). (F) Homozygous *Dl*^*attP*^*-Dl-NEQN2A-HA* flies die during embryogenesis and display the neurogenic cuticle phenotype. The arrow point to the small patch of remaining cuticle. (G-G’’) Notal region of a wing disc bearing *Dl*^*attP*^*-Dl-NEQN2A-HA* homozygous clones. The clones are labelled by the loss of GFP. (G’, G’’) magnification of the region highlighted by the arrows in (G). It reveals the neurogenic phenotype and the accumulation of Dl^attP^-Dl-NEQN2A-HA in the plasma membrane of the SOPs (arrow). This accumulation is much less obvious in the SOP developing in the heterozygous *Dl*^*attP*^*-Dl-NEQN2A-HA/+* region (G, G’, arrowhead).

## Data Availability

All constructs and fly lines generated for this work are available from the corresponding author upon request.
