# Supplementary figures and images for: The meaning of ubiquitylation of the DSL ligand Delta for the development of Drosophila
Source: BMC Biol. 2023 Nov 16;21:260. doi: 10.1186/s12915-023-01759-z (PMC10655352; doi:10.1186/s12915-023-01759-z)

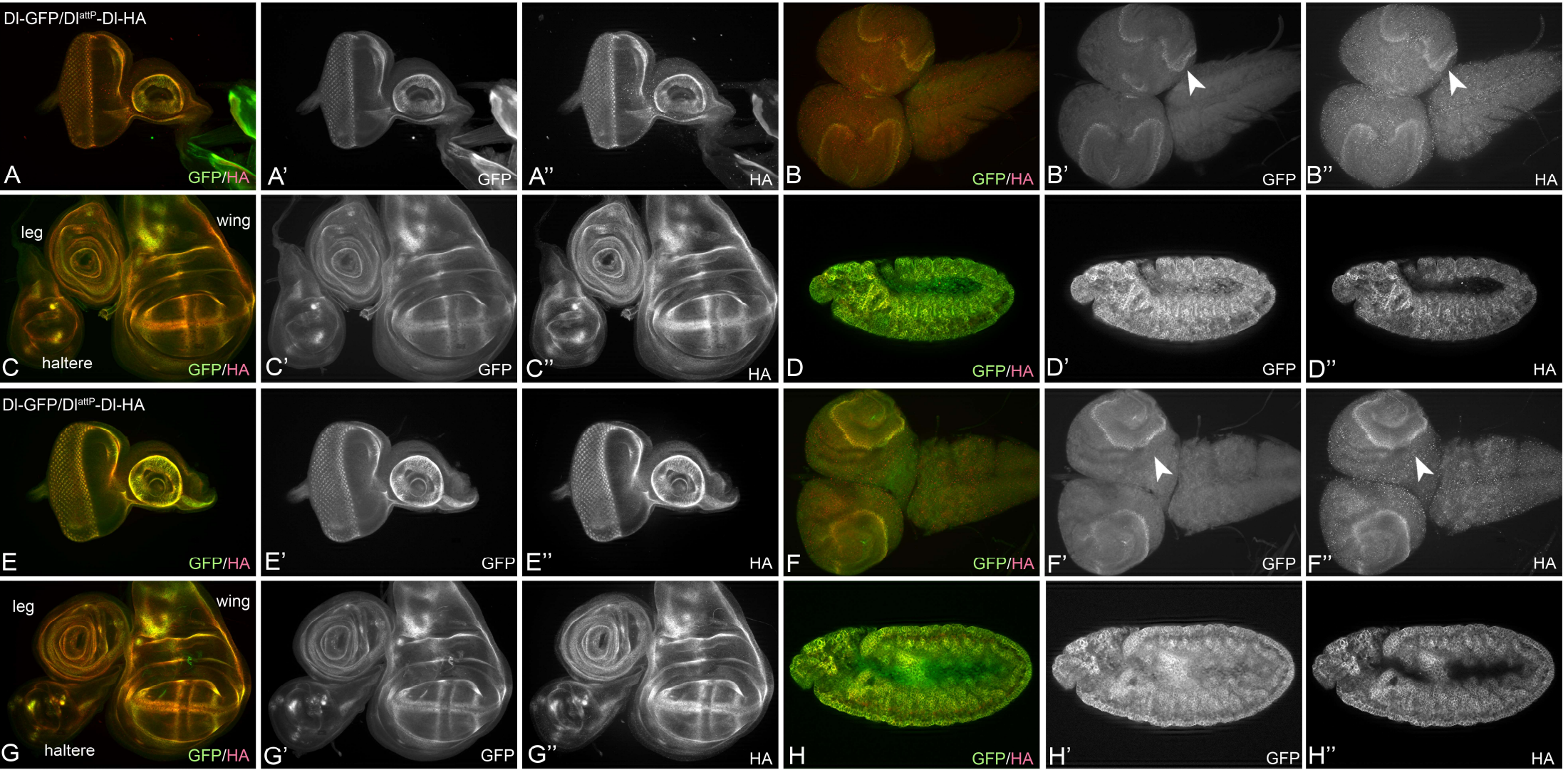

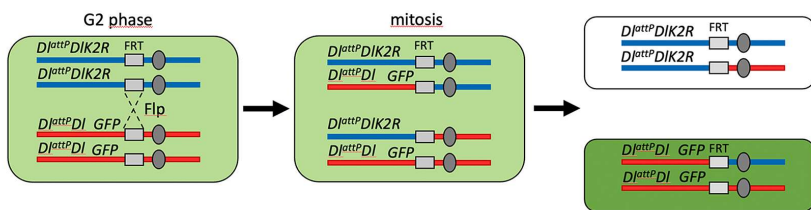

**A**

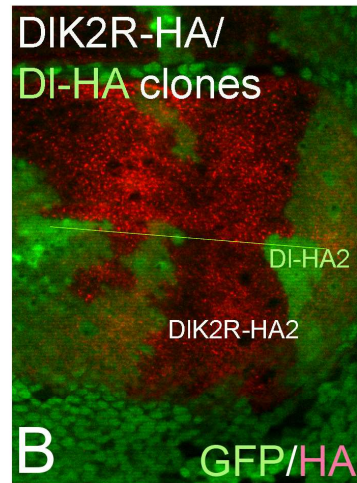

**B**

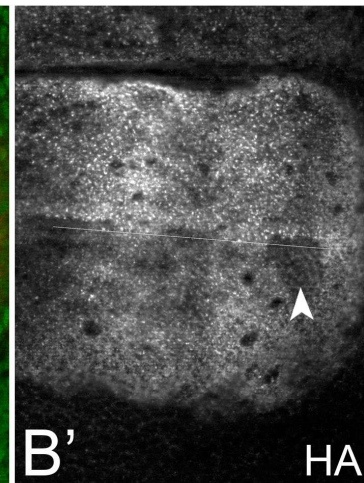

**B'**

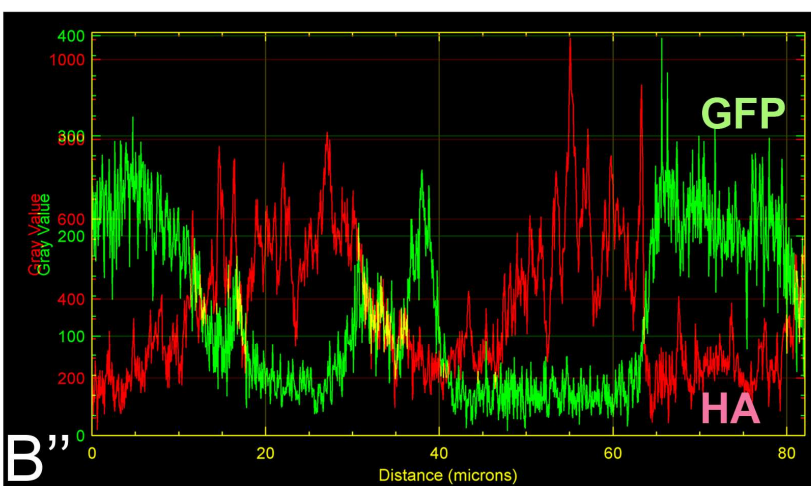

**B''**

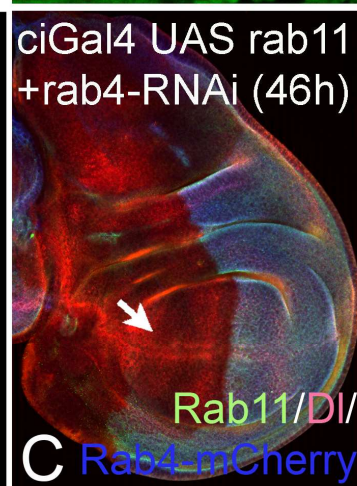

**C**

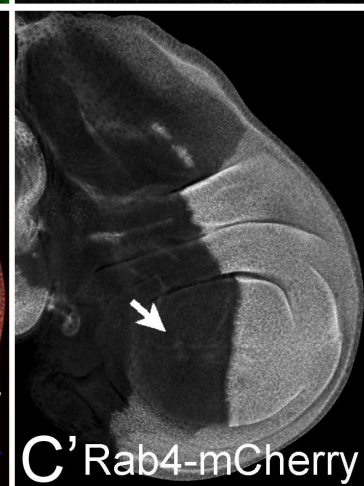

**C'**

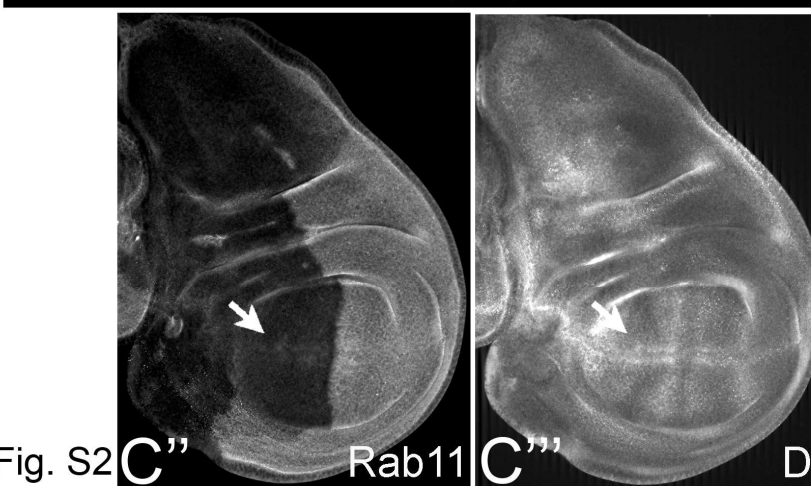

**C''**

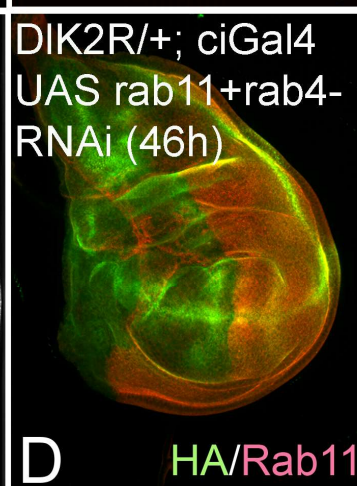

**D**

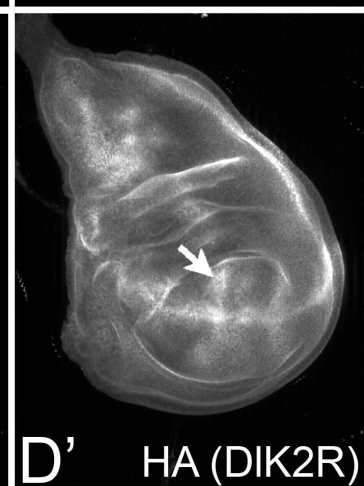

**D'**

Fig. S2

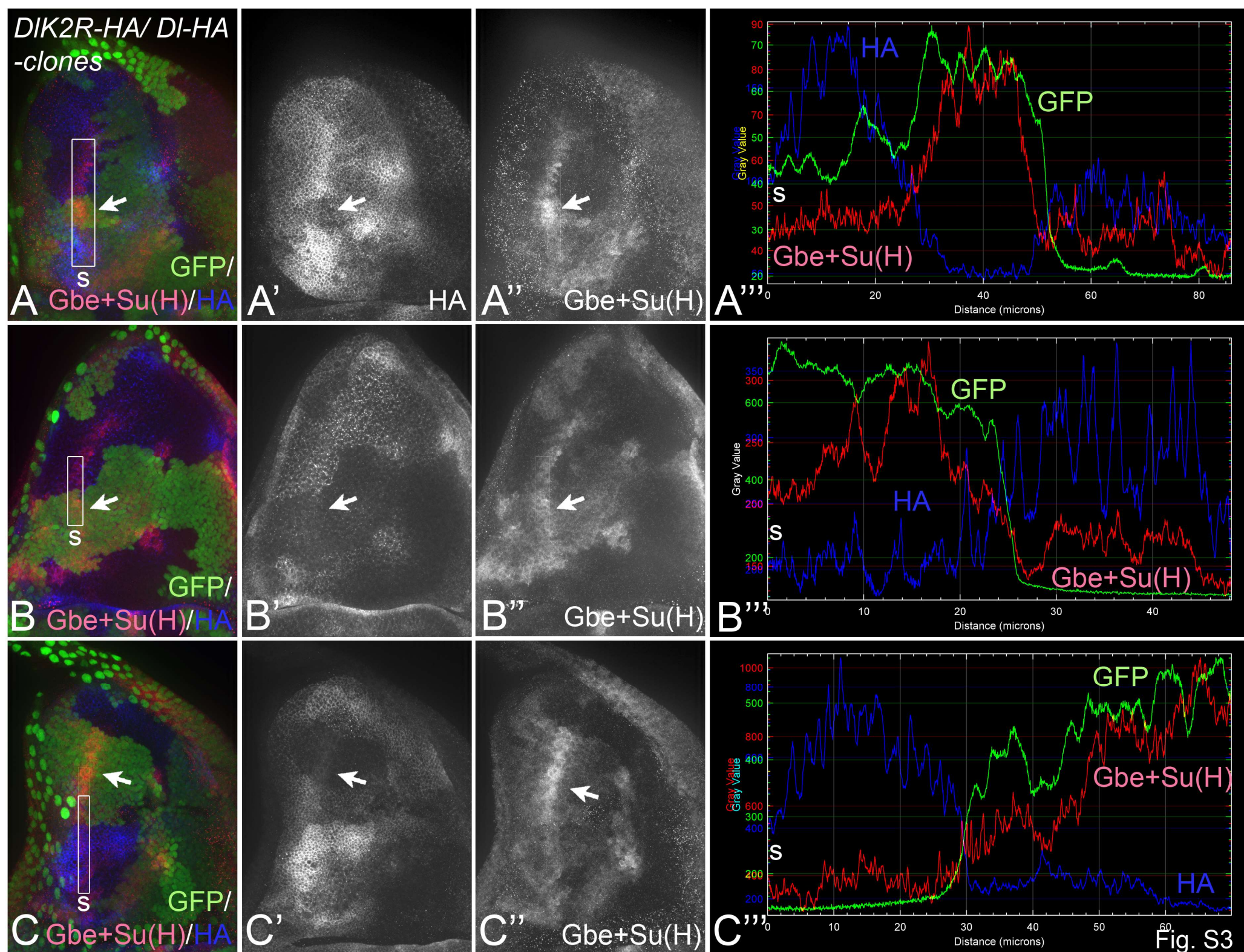

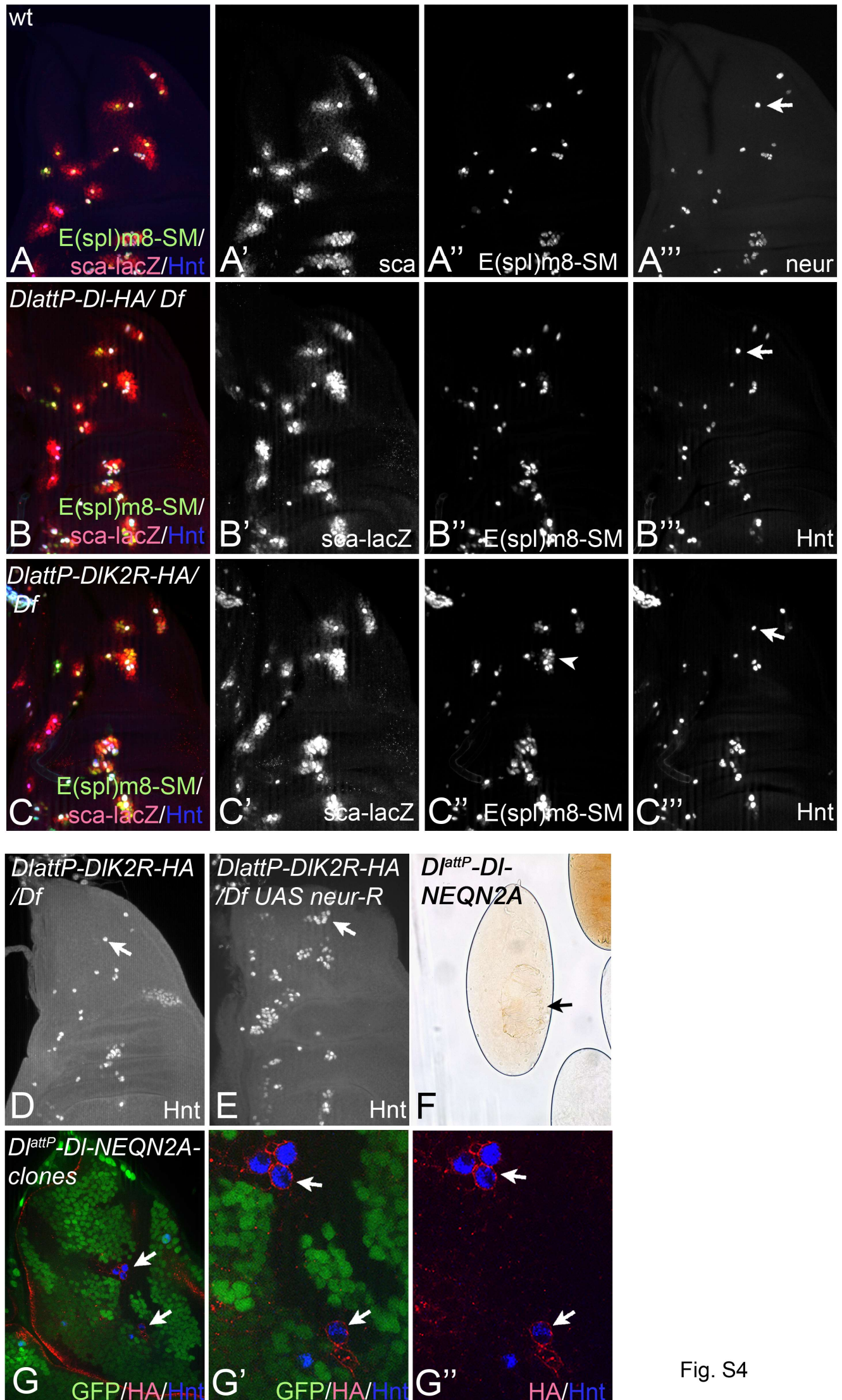

Fig. S4

Supplement: Supplementary file 1 — Additional file 1: Fig. S1. Expression of DlattP-constructs in various tissues compared to Dl::GFP. (A-A’’, C-C’’, F-F’’, H-H’’) Expression in various imaginal discs of the late third instar stage. (A-A’’, F-F’’) Expression in the eye imaginal disc. (C-C’’, H-H’’) Expression in the haltere, wing and leg disc.(B-B’’, G-G’’) Expression in the larval brain of the late third instar stage. The arrowhead highlights expression in the optic lobes. (D-D’’, I-I’’) Expression in stage 11 embryos. (E-E’’, J-J’’) Expression in the adult gut. Expression is largely restricted to escargot (esg-GFP) positive ISCs (E’’, J’’). Fig. S2. (A-B’’) Comparison of expression of DlattP-Dl-HA and DlattP-DlK2R-HA on the cell surface. Clonal analysis was used to twin clone that express either of the DlattP-variants in homozygosity, as described in (A). (B, B’) An example of a disc bearing the clones. The arrowhead points to a Dl-HA homozygous clone. The abundance of Dl is much reduced in the cells of Dl-HA homozygous in comparison to cells of DlK2R homozygous or heterozygous clones. (B’’) Pixel density measurements of the channels in the apical membrane region highlighted by the thin line in (B, B’). It revealed that Dl-HA is significantly less abundant than DlK2R-HA in the membrane. (C-C’’’) Co-depletion of Rab4 and Rab11 by expression of RNAi constructs for 46h using a combination of ciGal4 and tubGal80ts. It efficiently suppresses the expression of a mCherry-Rab4 construct (C’), as well as Rab11 (C’’). (D, D’) Co-depletion of Rab4 and Rab11 had no effect on the expression of DlK2R-HA, suggesting that endosomal recycling is not involved in the increase of it abundance in the membrane. Fig. S3. Three additional examples of wing discs with twin clones homozygous for DlattP-DlK2R-HA or DlattP-Dl-HA crossing the region of stripe2 at different points. Fig. S4. (A-C’’’) Analysis of SOP formation in DlattP-DlK2R-HA/Df (B-B’’’) and DlattP-Dl-HA/Df (C-C’’’) wing discs. (A-A’’’) The expression of the us [file 12915_2023_1759_MOESM1_ESM.pdf]
